# Genome-wide analysis of the *auxin*/*indoleacetic acid* (*Aux/IAA*) gene family in allotetraploid rapeseed (*Brassica napus* L.)

**DOI:** 10.1186/s12870-017-1165-5

**Published:** 2017-11-16

**Authors:** Haitao Li, Bo Wang, Qinghua Zhang, Jing Wang, Graham J. King, Kede Liu

**Affiliations:** 1 National Key Laboratory of Crop Genetic Improvement, Huazhong Agricultural University, Wuhan, 430070 China; 2 Southern Cross Plant Science, Southern Cross University, Lismore, NSW 2480 Australia

**Keywords:** *Brassica napus*, *Aux/IAA* gene, Chromosome distribution, Gene duplication, Expression pattern, Auxin response

## Abstract

**Background:**

*Auxin*/*Indoleacetic acid* (*Aux/IAA*) genes participate in the auxin signaling pathway and play key roles in plant growth and development. Although the *Aux/IAA* gene family has been identified in many plants, within allotetraploid *Brassica napus* little is known.

**Results:**

In this study, a total of 119 *Aux/IAA* genes were found in the genome of *B. napus*. They were distributed non-randomly across all 19 chromosomes and other non-anchored random scaffolds, with a symmetric distribution in the A and C subgenomes. Evolutionary and comparative analysis revealed that 111 (94.1%) *B. napus Aux/IAA* genes were multiplied due to ancestral *Brassica* genome triplication and recent allotetraploidy from *B. rapa* and *B. oleracea*. Phylogenetic analysis indicated seven subgroups containing 29 orthologous gene sets and two *Brassica*-specific gene sets. Structures of genes and proteins varied across different genes but were conserved among homologous genes in *B. napus*. Furthermore, analysis of transcriptional profiles revealed that the expression patterns of *Aux/IAA* genes in *B. napus* were tissue dependent. Auxin-responsive elements tend to be distributed in the proximal region of promoters, and are significantly associated with early exogenous auxin up-regulation.

**Conclusions:**

Members of the *Aux/IAA* gene family were identified and analyzed comprehensively in the allotetraploid *B. napus* genome. This analysis provides a deeper understanding of diversification of the *Aux/IAA* gene family and will facilitate further dissection of *Aux/IAA* gene function in *B. napus*.

**Electronic supplementary material:**

The online version of this article (10.1186/s12870-017-1165-5) contains supplementary material, which is available to authorized users.

## Background

Auxins were the first class of phytohormone to be discovered, and are prevalent signal chemicals produced within all vascular plants, and comprise a group of molecules with an indole ring. Auxins are able to regulate many aspects of plant growth and development, including cell division and elongation, as well as organ development at both cellular and whole plant level. They also play a role in regulating plant responses to environment such as phototropism, gravitropism, thigmotropism, and shade avoidance [1, 2]. This physiological regulation is achieved by changes in expression of many responsive genes resulting from auxin perception and signal transduction, including the well described transport inhibitor response 1/auxin signaling F-Box (TIR1/AFB) auxin signalling pathway [3]. Aux/IAA proteins are central repressors in this pathway and can interact with both TIR1/AFB and auxin response factors (ARFs). Aux/IAA proteins interact strongly with ARF activators, and may also have weak or no interaction with ARF repressors [4]. ARF activators induce the transcription of auxin responsive genes by an amino-terminal DNA binding domain (DBD) that binds to auxin response elements (AuxREs) on promoters of target genes [5]. Auxin signaling is regulated by a repression/de-repression mechanism with TIR1/AFB, Aux/IAA and ARF proteins in the TIR1/AFB pathway. At basal concentrations in plants, the heterodimer Aux/IAA-ARF represses expression of auxin-responsive genes [4, 6, 7]. Increased auxin can induce the ubiquitin–ligase complex SCF^TIR1/AFB^, which targets Aux/IAA proteins for ubiquitination and degradation, thus allowing ARF homodimers to activate the transcription of auxin-responsive genes [4, 6, 7].

Canonical Aux/IAA proteins contain four highly conserved domains designated I, II, III, and IV [8, 9]. Recent molecular and crystallographic studies indicate that these domains contribute to distinct roles of repression, degradation or interaction. Domain I contains a conserved LxLxLx motif that can recruit TOPLESS (TPL)/TPL-related (TPR) corepressors [10, 11] and is responsible for the repression property of the proteins [12]. Domain II contains a conserved degron GWPPV motif that interacts with the SCF^TIR1/AFB^ complex [13, 14] and confers instability to the proteins in the presence of auxin [15]. Domains III and IV contain a carboxy-terminal PB1 (Phox and Bem1) domain that is also located in the carboxy-terminal of ARFs, and thus are responsible for homo- and hetero-dimerization between Aux/IAA and ARF proteins [4, 16].

The biological functions of Aux/IAA proteins have mostly be revealed by identification and characterization of numerous gain-of-function mutants, where gain-of-function for each was located within the GWPPV motif of domain II, which stabilizes the Aux/IAA proteins in the presence of auxin [7, 17–21]. In both monocots and dicots, analysis of these mutants revealed that the *Aux/IAA* genes are important in auxin-related plant growth, including embryogenesis, growth and development of many organs including root, hypocotyl, leaf, stem and flower, tropism and apical dominance [7, 17–21]. For example, the *IAA1* locus in *Arabidopsis* is involved in root and shoot tropisms and development of diverse organs such as apical hook, rosette leaves, inflorescence and seeds [22]. In rice, *OsIAA11*, *OsIAA13* and *OsIAA23* each regulate initiation of lateral roots as well as development of aerial organs contributing to traits such as plant height and tiller number [18–20]. In addition, *Aux/IAA* genes are also involved in drought tolerance [23], nodule formation [24], and mediating the interaction of auxin and signaling of other hormones such as abscisic acid [25], cytokinin and ethylene [26, 27]. Moreover, the phenotypes of mutants associated with distinct members are similar but not identical, indicating that the functions of *Aux/IAA* members overlap but display their own specificity in plants. For example, in *Arabidopsis*, *IAA12* and *IAA16* each uniquely regulates early embryogenesis and fertility, respectively, although both control stem elongation, shoot apical dominance and leaf shape [25, 28, 29].

The *Aux/IAA* genes were first isolated from cDNAs induced by auxin in soybean [30], and orthologs were subsequently identified in the *A. thaliana* genome using PCR-based and yeast two-hybridization approaches [8, 31]. In the past ten years, the release of reference genomes for different species has provided an opportunity for genome-wide identification of *Aux/IAA* gene families using bioinformatics approaches. *Aux/IAA* gene families have been isolated and analyzed from many diverse plant species, including not only *A. thaliana* (*Brassicaceae,* 29 members) [32], but also the forest tree *Populus* (35 members) [33]; cereal crops such as rice (31 members) [34], sorghum (26 members) [35], maize (31 members) [36] and wheat (84 members) [37]; legume crops such as *Medicago truncatula* (17 members) [24], chickpea (22 members) and soybean (63 members) [38]; and some vegetable/fruit crops such as tomato (26 members) and potato (27 members) [39], cucumber (27 members) [40] and the diploid *Brassica rapa* (55 members) [41].

The *B. rapa* A genome comprises 10 of the 19 chromosomes of *B. napus* (canola, oilseed rape, rapeseed), which is one of the most important oil crops in the world. The diploid *Brassica* genomes arose following whole genome triplication (WGT) from an ancestor in common with *A. thaliana* ~15.9 million years ago (MYA). This was followed by divergence of *B. rapa* (AA, 2n = 20) from *B. oleracea* (CC, 2n = 18) ~4.6 MYA [42]. The allopolyploid *B. napus* (AACC, 2n = 38) formed ~7500 years ago, most likely in domestication, by hybridization between *B. rapa* and *B. oleracea* [43]. To date, little is known about the *Aux/IAA* gene family in *Brassica* species, and none of the *Aux/IAA* genes have been isolated and functionally analyzed from mutants of allotetraploid *B. napus*. In 2014, the draft genome of *B. napus* of cultivar Darmor-‘*bzh*’ was released, generated by both Sanger and next generation sequencing [43], which provided a good opportunity for genome-wide identification and analysis of the *Aux/IAA* genes in *B. napus*.

Given the significant importance of *Aux/IAA* genes in plant development, the objectives of this study were to: (1) comprehensively identify and map the *Aux/IAA* genes in the *B. napus* genome by sequence similarity; (2) analyze the nature of duplication events and phylogenetic relationships of the *Aux/IAA* gene family using the orthologous Aux/IAA proteins in *B. napus* compared with the related species *A. thaliana*, *B. rapa* and *B. oleracea*; (3) investigate and compare the gene structure and protein composition of *Aux/IAAs*; (4) profile the *Aux/IAA* gene expression pattern in four major tissues and determine differential expression under auxin treatment using RNA-seq data. These results will provide useful information for further studies in *B. napus* and other crops to unravel the functional involvement of the *Aux/IAA* family in diverse growth and development processes.

## Methods

### Plant materials and auxin treatment

Seeds of *B. napus* were sterilized, rinsed three times with sterile water and sown on Murashige and Skoog (MS) culture medium with 1% agar. The seedlings were grown at 22 °C with 16 h light/8 h dark. In addition, seeds of rapeseed were sown in the field to collect tissues of root, leaves, stem and flowering bud. For the IAA treatment, two sets of 30-day-old seedlings were incubated in liquid MS medium with and without 1 μM IAA for three hours, respectively. Each of ten seedlings for the both treatment were then pooled. All materials were frozen in liquid nitrogen and subsequently stored at −80 °C until RNA isolation.

### Identification of *Aux/IAA* genes

All protein sequences of *B. napus* cultivar ‘Darmor-*bzh*’ were downloaded from the Genoscope Genome Database (http://www.genoscope.cns.fr/brassicanapus/). Initially, these were searched against the Pfam library of Hidden Markov Model (HMM) profiles (http://pfam.xfam.org/) using hmmer3 software locally (http://hmmer.org/). The genes corresponding to proteins with the Aux/IAA domain (PF02309) were extracted from the *B. napus* genome and genes with both ARF and Aux/IAA domains were removed. Meanwhile, all proteins of *B. napus* were searched again via BLASTP algorithms with E value lower than 1e-10 using 29 *A. thaliana* Aux/IAA protein sequences as queries [32]. All obtained non-redundant protein sequences were then checked for the presence of the Aux/IAA domain by Conserved Domain (CD) search service on the NCBI website (https://www.ncbi.nlm.nih.gov/). Combining all of the results from this analysis, we identified all members of *Aux/IAA* genes in the currently available *B. napus* genome. To investigate the duplication and evolution profile of *Aux/IAA* genes in the *B. napus* genome, all members of *Aux/IAA* genes in the *B .oleracea* 'C' genome, one of the two diploid progenitor genomes of *B. napus*, were obtained using the same workflow as indicated for *B. napus*. For these, the protein sequences of *B. oleracea* (TO1000) were downloaded from the Ensembl Plants database (http://plants.ensembl.org/index.html). The *Aux/IAA* genes in *B. rapa* 'A' genome, the other diploid progenitor of *B. napus*, were obtained from a previous study [41].

### Chromosome mapping and orthologous identification of *Aux/IAA*

All *Aux/IAA* genes were mapped to specific *Brassica* chromosomes according to the location information retrieved from relevant GFF files. These *Aux/IAA* genes were allocated a unique name according to the functional gene nomenclature for the *Brassica* genus [44], based on their position on each chromosome. To investigate the whole genome duplication of *Aux/IAA* genes, most orthologous sets of *Aux/IAA* genes among *B. rapa*, *B. oleracea*, *B. napus* and their putative orthologs in *A. thaliana* were extracted from a previous study of the *B. napus* genome [43], and others were established by reciprocal BLASTP analysis. All *Aux/IAA* genes and their whole genome duplication distribution were displayed by Circos software [45].

### Gene structure, motif scanning and phylogenetic analysis of *Aux/IAA* genes

To illustrate the exon/intron composition of *Aux/IAA* genes, the structures of *Aux/IAA* genes were displayed using Gene Structure Display Server (GSDS 2.0) software (http://gsds.cbi.pku.edu.cn/index.php) using the gene annotation described in the GFF3 format. The physical and chemical parameters of each protein were calculated using ProtParam (http://www.expasy.ch/tools/protparam.html). Motifs of the Aux/IAA proteins were investigated with the MEME tool (http://meme-suite.org/index.html). Default parameters were used, apart from the number of found motifs was set as four, with motif width ranging from 6 to 60. All conserved domains and signal peptide were investigated based on multiple sequence alignment of Aux/IAA proteins performed by Cluster Omega program (http://www.ebi.ac.uk/Tools/msa/clustalo/), and displayed by Jalview 2.0 [46]. Phylogenetic relationships were established using MEGA 5.2 [47] by the Neighbor-Joining (NJ) method based on *p*-distance model of amino acid substitutions type. A non-parametric bootstrap method was performed with bootstrap replication of 1000.

### Motif analysis in the promoter regions of *Aux/IAA* genes

To identify the *cis*-elements in the promoter region of each Aux/IAA gene, 2000 bp of genomic sequence upstream of the translation start site was retrieved from the available *B. napus* genome sequence and analyzed by PlantPAN 2.0 (http://plantpan2.itps.ncku.edu.tw). However, for genes where the length of promoter was less than 2000 bp, the inter-genic sequence up to the neighboring upstream gene was extracted for analysis.

### Transcriptome analysis based on RNA-seq data

To reveal the expression profile of *Aux/IAA* genes in *B. napus*, RNA-seq data from major tissues, including roots, stem, leaves and flower buds, were obtained from a previous study [43]. All reads were mapped to the *B. napus* ‘Darmor-*bzh*’ genome v4.2 using HISAT2 (v2.0.4). Fragments per kilobase per million mapped reads (FPKM) were calculated using Cufflinks (v2.2.1) to estimate gene expression levels. Heat maps were constructed using R package pheatmap based on normalized expression values of *Aux/IAA* gene. For the auxin response of *Aux/IAA* genes, the RNA integrity of indicated samples was determined with an Agilent 2100 Bioanalyzer (Agilent Technologies) and RNA concentration was measured using a Qubit®2.0 (Life Technologies). The libraries were constructed according to TruSeq® RNA Sample Preparation v2 Guide (Illumina) and sequenced on the HiSeq3000 platform. Read mapping and FPKM calculation were performed as described above. Differential expression genes (DEGs) were identified using the R package DEGseq based on the read count for each gene with thresholds that adjusted *p* value (*q* value) < 0.05 and an absolute value of log_2_(fold change) ≥ 1.

### qRT-PCR analysis

Total RNA was extracted using the ultrapure total RNA isolation kit (BioTeke, Beijing). The quality was checked on 2% agarose gel and concentration was quantified in a spectrophotometer. Genomic DNA was removed by digestion with DNase I and the first strand cDNA was synthesized from total RNA according to the manufacturer’s instructions (Thermo Scientific). cDNA was diluted 10-fold as the templates of RT- PCR. The qRT-PCR reactions were performed on a CFX96 Touch Real-Time PCR detection system (Bio-Rad) using SYBR Green Supermix (Bio-Rad). Each sample was represented by three biological and two technical repeats. The PCR reaction and cycling protocol were carried out according to manufacturers' instructions. Melting curves were generated from 65 to 95 °C with 0.5 °C increments at 5 s/step to estimate the specificity of product. The expression level of target genes was analyzed following deltaCt method with *B. napus ENTH* gene as reference for normalization [48]. Primers for qRT-PCR were listed in Additional file 1.

## Results

### Genome-wide identification and chromosomal distribution of *Aux/IAA* genes in *B. napus*

In order to identify all the *Aux/IAA* gene members in *B. napus*, 101,040 available protein sequences were obtained from the *B. napus* database. 182 non-redundant genes were extracted as candidate *Aux/IAA* members by a Hidden Markov Model (HMM)-based search with Aux/IAA domain (PF02309) and BLASTP with 29 *A. thaliana Aux/IAA* genes as queries. Of these, 58 genes were removed that contained additional B3 and ARF domains characteristic of the ARF gene family. After checking for the presence of Aux/IAA domains using the Conserved Domain (CD) search platform, a total of 119 *Aux/IAA* members were identified in the *B. napus* genome (details in Additional file 2).

**Fig. 1:**
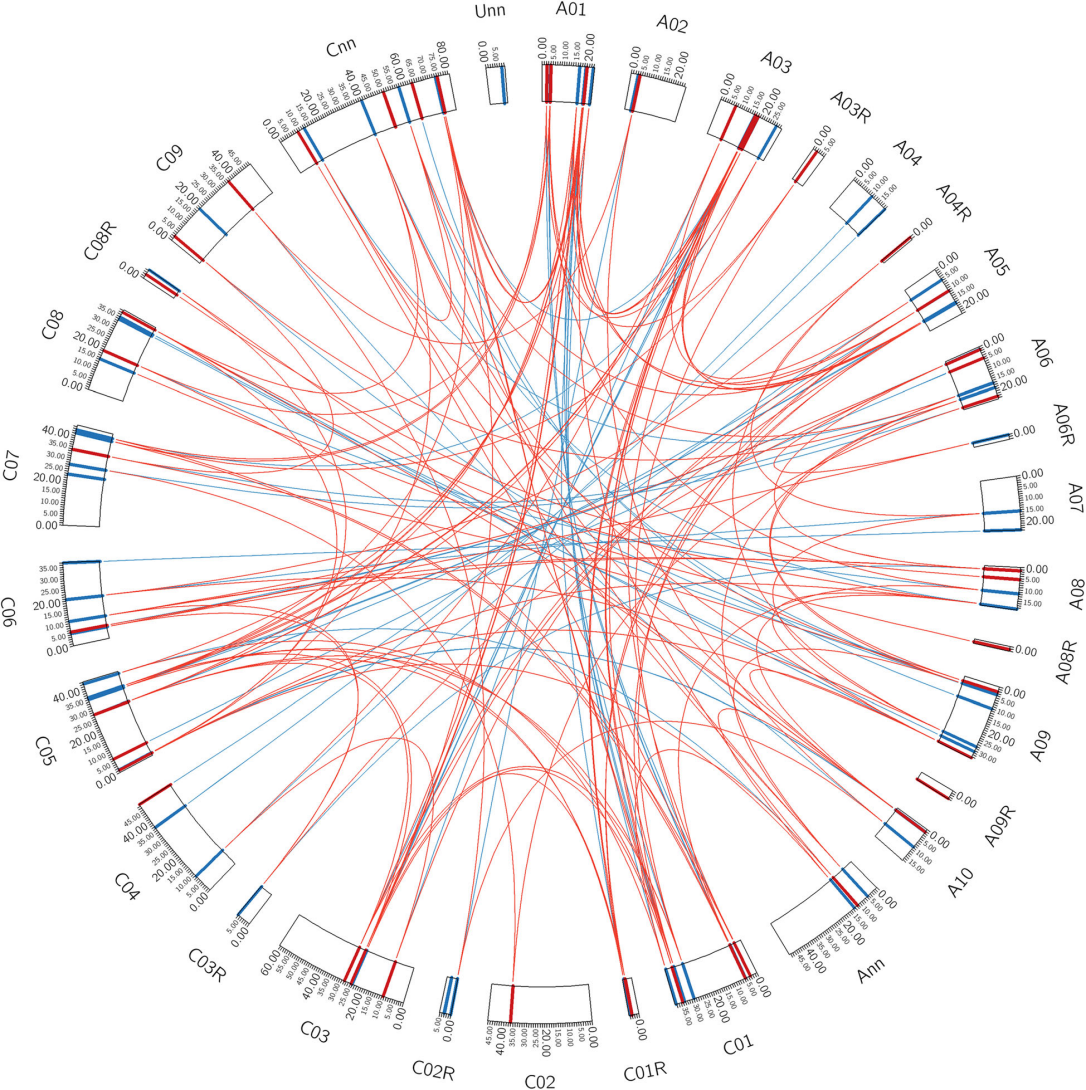
Distribution and duplicated nature of *Aux/IAA* genes in *B. napus* genome. Outer boxes represent chromosomes in the *B. napus* genome. Colored lines in boxes indicate location of Aux/IAA genes in each chromosome, where blue and red lines show forward and reverse transcription direction, respectively. The duplicated gene pairs resulting from *Brassica* WGT and recent allotetraploidy are linked by red and blue lines, respectively

The chromosomal location and direction of transcription for each *Aux/IAA* gene were established, with 94 positioned on the 19 chromosomes and 25 located on random scaffolds of the ‘Darmor-*bzh*’ reference sequence [43] (Fig. 1). We analyzed the distributions of the 118 *Aux/IAA* genes across the genome of *B. napus*, excluding *BnaX.IAA.1* which lacked location information on an unassigned scaffold. The *Aux/IAA* genes have a non-random distribution across the 19 chromosomes, but are equally distributed on the A and C subgenomes (57 and 61 genes, respectively) (Fig. 1; Additional file 2). The number of *Aux/IAA* genes in the A and C subgenomes appears almost identical to that in the ancestor genomes *B. rapa* (Ar genome, 55) [41] and *B. oleracea* (Co genome, 60, Additional file 3). The number of *Aux/IAA* gene varied dramatically between chromosomes, with a minimum of two on chromosome A07 and a maximum of ten on chromosomes C01 and C05 (Fig. 1; Additional file 2). In addition, three and eight *Aux/IAA* genes were present on unassigned scaffolds in the A subgenome and the C subgenome, respectively. Five tandem *Aux/IAA* gene pairs located on chromosomes A03, A10, C03, C05 and C08 (Fig. 1). Similar tandem pairs have been observed in the *Populus*, chickpea and soybean genomes [33, 38]. Notably, for the tandemly duplicated genes *BnaC03.IAA.4* and *BnaC03.IAA.5* on chromosome C03, *BnaC03.IAA.5* may represent a recently truncated gene generated by gene conversion between the extensively homoeologous A3 and C3 chromosomes (Additional file 2). This is supported by the phylogenetic distance of the non-homeologous pair of *BnaA03.IAA.5*/*BnaC03.IAA.5* which was closer than that of homeologous pair *BnaA03.IAA.5*/*BnaC03.IAA.4* that resulted from the recent allotetraploidy of *B napus* (Additional file 2).

### Comparative analysis of *Aux/IAA* genes in *Brassica* species and their duplication in *B. napus*

The evolution and duplication of *Aux/IAA* genes in *Brassica* was analyzed using gene models from genomes of *A. thaliana*, *B. napus* (An and Cn genome) and its diploid progenitors *B. rapa* (Ar genome) and *B. oleracea* (Co genome). Previous analyses identified 53 of the 55 *Aux/IAA* genes in the *B. rapa* genome to be orthologs corresponding to 29 *A. thaliana* genes [41], compared with 55 of the 60 *Aux/IAA* genes in *B. oleracea* (Additional file 3). Most of the *Arabidopsis Aux/IAA* genes had one or two orthologs in the *B. rapa* and *B. oleracea* genomes. However, seven *A. thaliana Aux/IAA* genes each had three orthologs in both *B. rapa* and *B. oleracea* genomes, respectively, and another two *A. thaliana Aux/IAA* genes had three orthologs only in the *B. oleracea* genomes (Additional file 3), which indicated that most *Aux/IAA* genes experienced gene loss after WGT. Compared to *B. rapa* and *B. oleracea*, 50 (87.7%) and 53 (86.8%) orthologous *Aux/IAA* gene pairs were observed between the An and Cn subgenomes and their respective progenitor genomes (Additional file 3). Most of the orthologous *Aux/IAA* gene pairs (43, 84.3%) between ancestors *B. rapa* and *B. oleracea* remain as homeologous pairs in *B. napus* (Fig. 1; Additional file 3). This retention rate of duplication from ancestor is almost same as the rate for all homeologous gene pairs across the whole *B. napus* genome (*p* = 1.0), where 27,360 out of 32,699 orthologous gene pairs (83.7%) between *B. rapa* and *B. oleracea* were conserved as pairs of homeologous genes in *B. napus* [43]. These observations suggest that most of the *Aux/IAA* genes were retained intact during the recent formation of the allotetraploid *B. napus* from *B. rapa* and *B. oleracea*. In summary, homologous analysis of *Aux/IAA* genes revealed that as a result of both *Brassica* WGT and recent allotetraploidy, 111 different *Aux/IAA* gene family members were represented by two to six copies each on different chromosomes of the *B. napus* genome, with the remaining eight *Aux/IAA* genes being unique (Fig. 1).

### Phylogenetic relationship of aux/IAA genes in *Arabidopsis* and *Brassica*

**Fig. 2:**
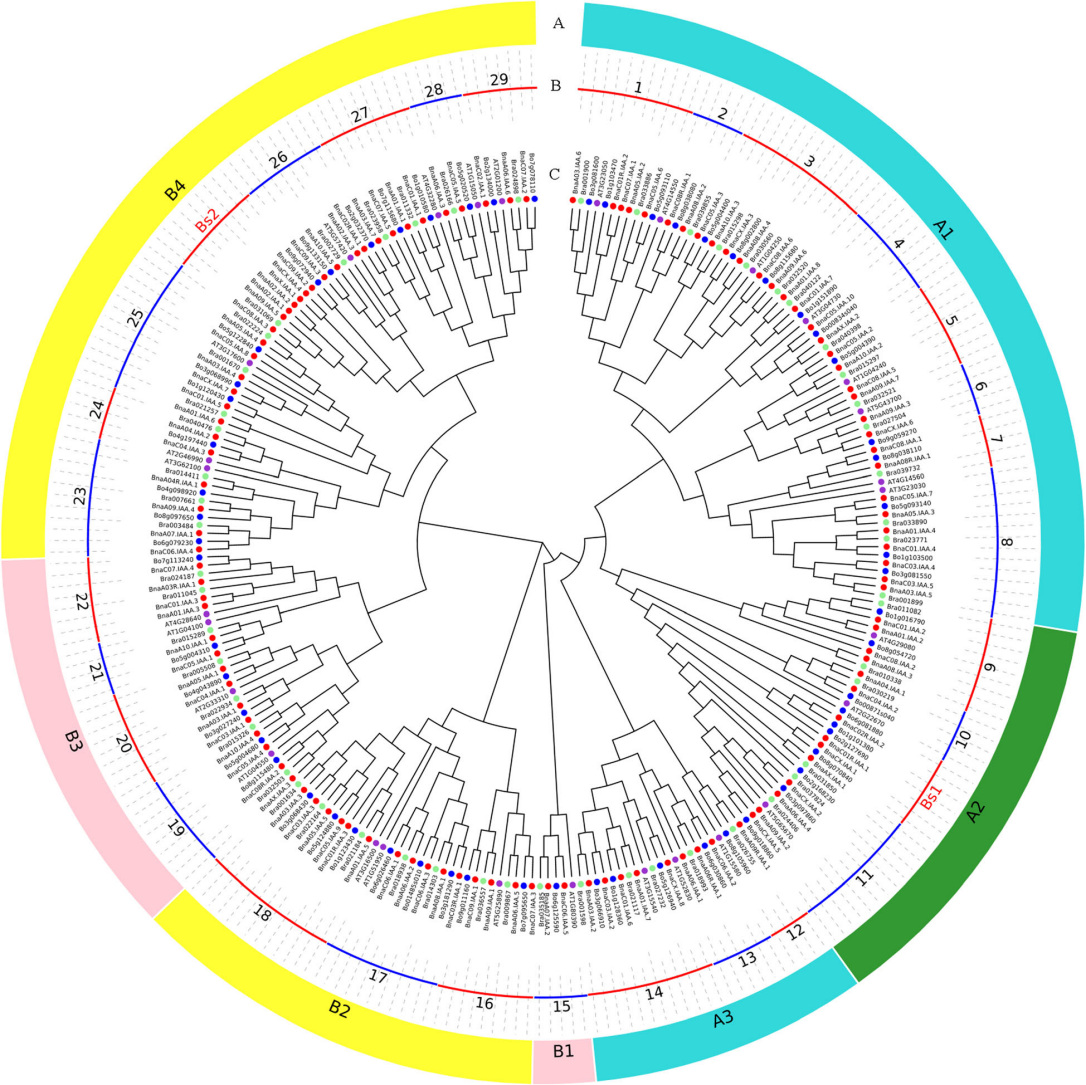
Phylogenetic relationship of *Aux/IAA* genes among *A. thaliana* and *B. rapa*, *B. oleracea*, *B. napus*. The unrooted tree was generated using MEGA5.2 based on alignment of full-length amino acid sequences of 29 *A. thaliana*, 54 *B. rapa*, 60 *B. oleracea* and 118 *B. napus* Aux/IAA proteins. **a** Seven subgroups were displayed by colored arcs; **b** The 29 orthologous sets and two *Brassica* specific sets are indicated with colored lines; **c** The *Aux/IAA* genes from *A. thaliana* and *B. rapa*, *B. oleracea*, *B. napus* were indicated by yellow, lightgreen, blue and red dots, respectively

To examine the evolutionary relationships among the *Aux/IAA* genes from *B. napus, B. rapa, B. oleracea*, and *A. thaliana,* a rooted phylogenetic tree was generated based on the alignment of amino acid sequences for 261 *Aux/IAA* genes, including 29 *A. thaliana*, 54 *B. rapa*, 60 *B. oleracea* and 118 *B. napus* members. *BnaC08.IAA.4* could not be clustered and was removed from further analysis. The phylogenetic tree could be divided into Group A and Group B, which could further be divided into three (A1-A3) and four (B1-B4) subgroups respectively (Fig. 2). This pattern of two major groups for *Aux/IAA* gene family members in the phylogenetic tree was similar to that reported for other plants including the monocots wheat [37], maize [36] and rice [34], and the dicots tomato [39] as well as *B. rapa* [41], which suggests that the *Aux/IAA* genes have been widely conserved in different taxa.

Group A and B consisted of 125 and 136 *Aux/IAA* genes, respectively. Twenty nine orthologous gene sets were identified with *A. thaliana Aux/IAA* genes as a reference and distributed in all seven subgroups (Fig. 2). Sixteen *Brassica-*specific *Aux/IAA* genes, which did not have any orthologs in the *A. thaliana* genome (Additional file 3), were clustered into two sets in subgroup A2 and B4 and designated as Bs1 and Bs2, respectively (Fig. 2). This suggests that these two sets of *Brassica*-specific *Aux/IAA* genes may have similar functions. All subgroups except subgroup B1 included multiple sets of orthologs. It was apparent that all 29 orthologous sets grouped together in neighboring branches. A sister pair indicates the closest relatives within a phylogenetic tree. Within this tree, a total of 97 sister pairs were found, consisting of 45 and 52 pairs in group A and B (Fig. 2). Most of the sister pairs were orthologous *Aux/IAA* gene pairs between the An and Cn subgenomes and their respective progenitor genomes, with 43 An-Ar pairs and 47 Cn-Co pairs. These two observations add further support to the results of the *Aux/IAA* gene duplication analysis. For two-thirds of the orthologous sets where two or three copies of the *Aux/IAA* genes were present in the A/C genome, the evolutionary relationship between *A. thaliana* genes with one of the *Brassica* orthologs derived from the genome triplication was closer than between the orthologs themselves (Fig. 2). This phenomenon has also been observed for other genes [49] and is consistent with the two steps of WGT leading to the *Brassica* subgenomes.

### Gene and protein structure of IAAs in *B .napus*

**Fig. 3:**
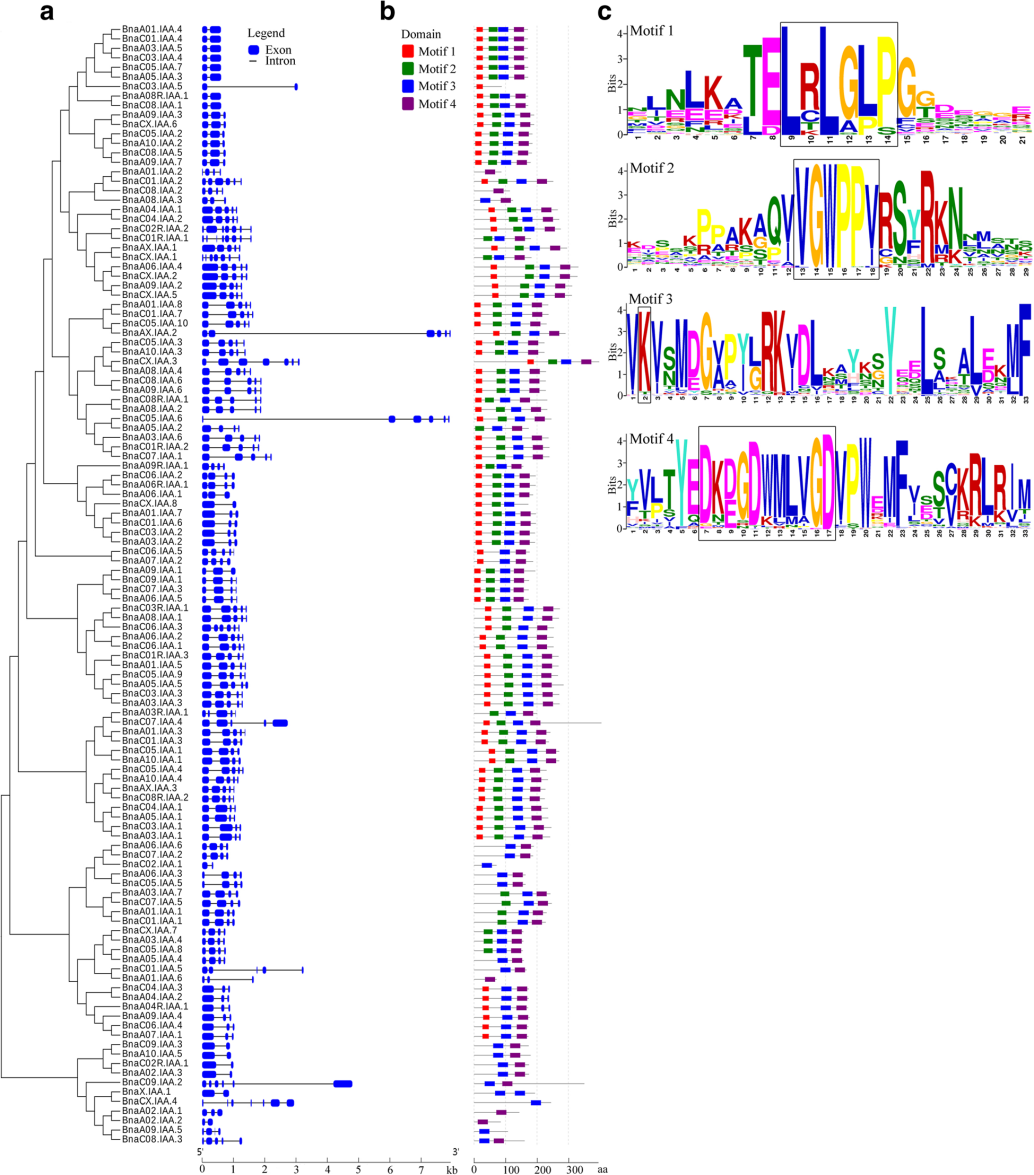
Gene and conserved motif structure of the *B. napus Aux/IAA* gene family. **a** Exon-intron organization of *B. napus Aux/IAA* genes. The blue solid boxes represent exons and black lines represent introns. **b** Conserved motif analysis of *B. napus* Aux/IAA proteins. The motifs representing four conserved domains are marked in red, green, blue and purple solid boxes, respectively. **c** The amino acid sequence of four motifs. The bits indicate amino acid conservation in each positon. LxLxLx motifs in domain I, GWPPv/i motifs in domain II and conserved lysine and the OPCA-like motif phosphorylation sites in domain III/IV are highlighted with black boxes

The open reading frame (ORF) length of the 118 *Aux/IAA* genes ranged from 340 to 7942 bp, with an average of 1336 bp. This corresponded to the coding domain sequence (CDS) length of the 118 *Aux/IAA* genes which ranged from 216 to 1213 bp, with an average of 632 bp (Additional file 2). This suggests that variation of intron length was more extensive than that of flanking exons as seen in the schematic diagram of the genes (Fig. 3a). The number of exons varied from two to eight, with most genes (108, 91.5%) having two to five exons. The distribution of exons and introns is complex, with a different structural pattern of exon/intron composition even within the same phylogenetic subgroup. However, the homologous genes have a similar pattern of gene structure (Fig. 3a; Additional file 4). We therefore take the 17 homologous gene sets in *B. napus* that have an identical exon number as in *B. rapa*, *B. oleracea* and *A. thaliana* to correspond to ancestral genes, with the remainder having only one or two differences (Fig. 3a; Additional file 4). Moreover, 71.7% (124/173) of the homologous *Aux/IAA* gene pairs in the *B. napus* genome had an almost similar structural pattern with respect to exon number and CDS length, which was a higher proportion than for all paralogous gene pairs reported in the ancestral *B. rapa* and *B. oleracea* genome [42]. This observation indicates that the *Aux/IAA* gene families may be more conserved in *Brassica*, possibly due to their importance in facilitating plasticity of plant development.

The various physical and chemical properties of Aux/IAA proteins, including polypeptide length, molecular weight, instability index and Grand average of hydropathicity (GRAVY), were calculated (Additional file 2). The polypeptide length ranged from 72 to 405 aa, with molecular weight ranging from 8.39 to 44.47 kD. The GRAVY index of all proteins was negative, indicating that all Aux/IAA proteins in *B. napus* are hydrophilic. Seventy seven (65.3%) Aux/IAA proteins had an instability index of more than 40, and may be unstable in vitro. Multiple amino acid alignment showed that there were four conserved regions designated as domain I, II, III and IV in *B. napus* Aux/IAA proteins (Additional file 5). Domain I contained an LxLxLx motif, an EAR-like repression motif recruiting TOPLESS (TPL) co-repressors. Domain II contained VGWPPV(I) motifs, the conserved degron of Aux/IAA protein in auxin signaling. Domain III and IV contained putative canonical PB1 domains, including both an invariant lysine typical of type II PB1 domains and a type I PB1 series of acidic residues (D-x-D/E-x-D-x_n_-D/E) [16], mediating interaction of ARF and Aux/IAA. Four conserved Aux/IAA protein domains were represented with four motifs generated from MEME analysis (Fig. 3b and c). Seventy five (63.6%) of the proteins contained four canonical domains, compared to 20 (16.9%) that contained three domains (eleven for II, III and IV; eight for I, III and IV; one for I, II and III). Thirteen (11.0%) proteins contained two domains (III and IV), with the remaining ten (8.5%) only having a single domain (five for IV; four for III; one for I). It is notable that the proteins belonging to the same subgroup have a similar domain distribution. Almost all Aux/IAA proteins in subgroup A1, A2, A3, B2 and B3 contain the canonical four domains, whereas all Aux/IAA proteins in subgroup B1 and B4 contain non-canonical domains (Additional file 6). However, some Aux/IAA proteins that belong to the same orthologous sets in subgroup A1 and A2 have truncated domains, such as BnaA05.IAA.2 in set 1, BnaC03.IAA.5 in set 8 and BnaA08.IAA.3, BnaC08.IAA.2 and BnaA01.IAA.2 in set 9. The truncated Aux/IAA proteins in subgroup A1 and A2 and B1 appear to have been formed after the divergence between *A. thaliana* and *Brassica*, since the orthologs in *A. thaliana* contained the canonical four domains (Additional file 6). In addition, the majority of Aux/IAA proteins in *B. napus* had two nuclear localization signals (NLSs), one being a bipartite NLS and the other a SV40-like NLS (Additional file 5). Interestingly, a subset of 20 Aux/IAA proteins appeared to contain a second repression domain (LxLxLx motif) between domain I and II, which also has repressive capacity in the auxin signaling pathway (Additional file 5) [50].

We assigned GO annotation to the 118 *Aux/IAA* genes to investigate the biological processes they potentially regulate (Additional file 7). Based on the cellular components, most of the genes were localized in cellular (organelle), consistent with the NLS identification based on amino acid alignment. Based on biological process, most of genes participated in response to stimulus, cellular process, pigmentation, biological regulation and metabolic process. In addition, some genes were also specifically involved in other biological process, such as reproduction process and immune system process. These results suggest that *Aux/IAA* genes have acquired multiple biological roles in *B. napus*. Based on molecular function, most of the *Aux/IAA* genes had binding and transcription regulator activity, which was consistent with their role as repressors in auxin signaling pathway.

### Expression patterns of *Aux/IAA* genes in *B. napus*

**Fig. 4:**
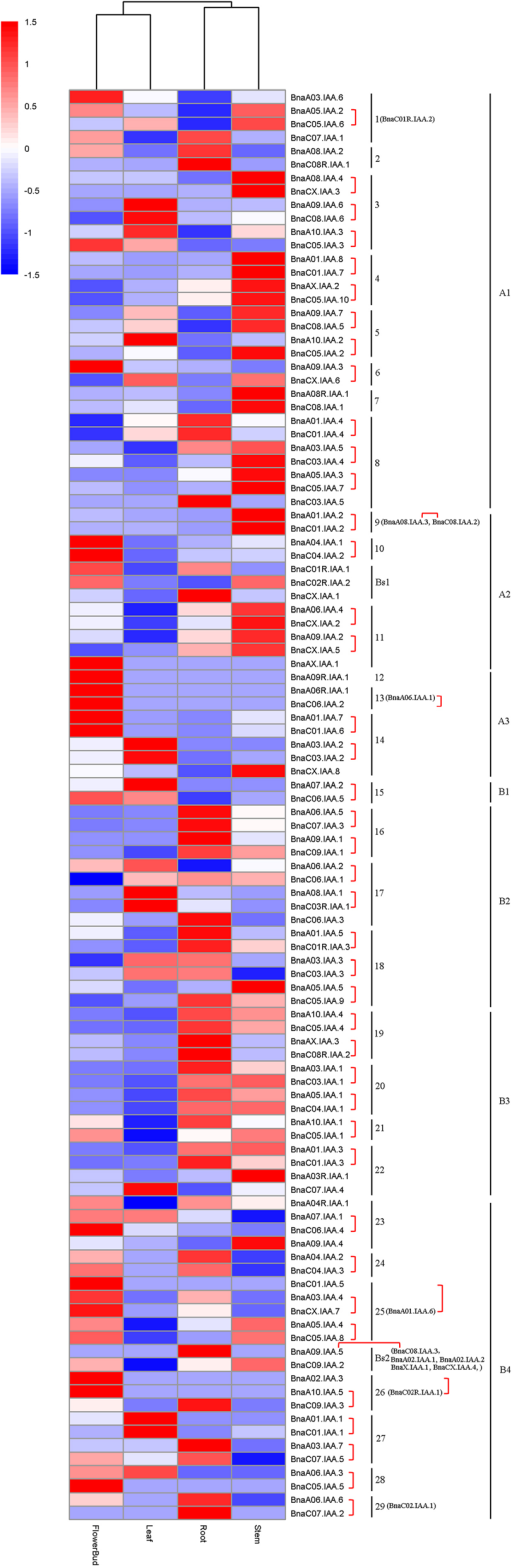
Heatmap of expression profiles of *B. napus Aux/IAA* genes. The design of subgroup and homologous sets is as shown in Fig. 2. The homeologous genes between An and Cn genomes resulting from recent allotetraploidy are highlighted by red square brackets

To gain insights into the putative functions of *Aux/IAA* genes in plant development, we analyzed their expression patterns in four major tissues including roots, stem, leaves and flower buds based on RNA-seq data. A subset of 12 genes lacked expression in any of these four tissues (Fig. 4; Additional file 8), of which almost half were clustered in the Bs2 group. This suggests that most of the *Brassica-*specific genes might be non-functional or have inducible expression. The RNA-seq data indicated that the expression of *Aux/IAA* members was highly variable among tissues, supporting the diversification of functions for the *Aux/IAA* genes during *B. napus* development. Cluster analysis showed that a similar expression pattern was observed between flowering bud and leaf, root and stem, (Fig. 4). Twenty four *Aux/IAA* genes had tissue(s)-specific expression patterns (Fig. 4, Additional file 8), although most of these had a very low expression level (FPKM <1), suggesting that these genes may be less important for growth and development in *B. napus*. We validated six *Aux/IAA* genes (*BnaA04.IAA.1*, *BnaC04.IAA.2*, *BnaA06.IAA.6*, *BnaA09R.IAA.1*, *BnaAX.IAA.1* and *BnaC01.IAA.5*) by quantitative RT-PCR, and found that two of these (*BnaA04.IAA.1* and *BnaC04.IAA.2*) express in all four tissues, which is consistent with the RNA-seq data (Additional file 9). The expression level and specificity of the other four tissue(s)-specific genes was also consistent with the RNA-seq data (Additional file 9).

The phylogenetic analysis had generated seven subgroups with 29 orthologous sets. In general, *Aux/IAA* genes in the same orthologous sets had distinct expression patterns across different tissues, suggesting acquisition of subfunctionalization. However, most of the homeologous pairs (An and Cn) from the recent allotetraploidy shared a similar expression pattern across tissues, although some had a diverged pattern (Fig. 4). For example, within homologous set 3, the *BnaA08.IAA.4* and *BnaCX.IAA.3* homeologous pair from the allotetraploid exhibited a similar expression pattern, with higher transcription in stem. Likewise, another homeologous pair, *BnaA09.IAA.6* and *BnaC08.IAA.6*, were both highly expressed in leaf. In contrast, *BnaA10.IAA.3* and *BnaC05.IAA.3* were found to be differentially expressed across tissues, with a higher level in leaf and flower bud respectively, although they represented homologs within the allotetraploid (Fig. 4). Moreover, distinct expression patterns were observed among the subgroups. The majority of genes in subgroup A1, A2, A3, B1, B2 and B3 exhibited higher expression level across four tissues. In contrast, most of the genes in subgroup B4 exhibited lower or even no expression (Additional file 8).

### Expression analysis of *Aux/IAA* genes during seedling stage under auxin treatment

**Fig. 5:**
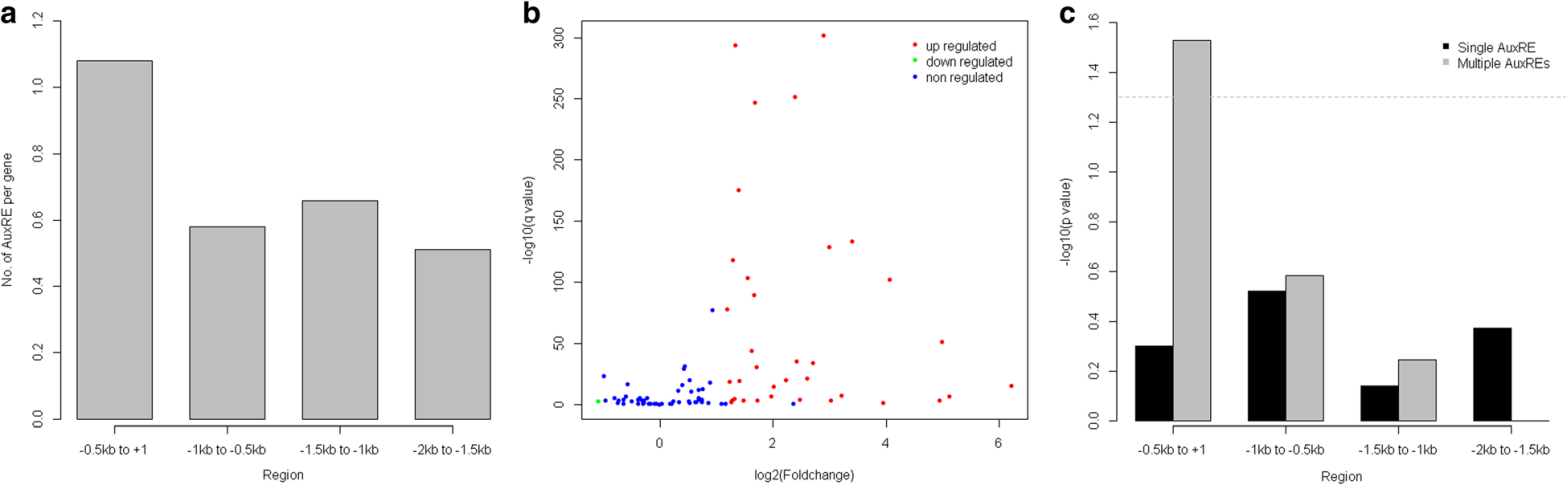
Analysis of AuxREs in promotors and auxin-induced expression of *B. napus Aux/IAA* genes. **a** Distribution of AuxREs in the 2.0 kb regions upstream of translation initiation site. The location of translation initiation site was designed as +1 bp and the numbers of AuxREs were calculated at intervals of 500 bp. **b** Scatterplot showing the significantly differentially expressed *Aux/IAA* genes. The fold change of *Aux/IAA* genes expression in auxin treated seedlings relative to control was expressed as log_2_(foldchange) on the x-axis. The adjusted *p* value calculated by DEGseq program was expressed as -log_10_(*q*-value) on the y-axis. **c** Enrichment analysis of auxin up-regulation of *Aux/IAA* genes. The associations between presence of AuxREs and auxin up-regulation were analyzed by Pearson’s chi-square test where a *p*-value <0.05 indicates a significant association. The horizontal dotted gray line indicates a threshold of –log_10_(*p* value) = 1.30

*Aux/IAA* gene members are one of the three major early auxin-inducible gene families, and many auxin-responsive *cis*-elements (AuxREs) have been found in promoters of these genes [9]. To find potential AuxREs, we performed a motif search in the 2 kb promoter region of all *B. napus Aux/IAA* genes. AuxREs were found in the promoter of 101 genes (85.59%), with AuxRE counts ranging from one to nine. Of these, 21 (17.80%) *Aux/IAA* genes contained single and 80 (67.79%) multiple AuxREs in the promotors. The distribution of AuxREs along the promotors is non-uniform, with a significantly higher frequency in the proximal promoter (−0.5 kb to +1) of the *Aux/IAA* genes (Fig. 5a). At the same time, we analyzed the expression of *Aux/IAA* genes under auxin treatment. A total of 34 genes was significantly up-regulated by exogenous auxin treatment (Fig. 5b), all of which had AuxREs in the promotors. A detailed analysis was performed to analyze the association between the presence of AuxREs and auxin responsiveness of *Aux/IAA* genes. Significantly up-regulated genes were enriched in those with multiple AuxREs in the proximal promoter region (Fig. 5c).

## Discussion

As a large distinct plant gene family, *Aux/IAA* genes regulate auxin-induced gene expression and diverse aspects of plant development via the Aux/IAA-ARF and Aux/IAA-TIR complex [3, 4]. Recent study in *A. thaliana* has shown that members of the *Aux/IAA* gene family have different properties. Firstly, Aux/IAA proteins differ in their capacity to interact with ARF activators in vitro, and most Aux/IAA-ARF interacting pairs have a diverse co-expression pattern that might lead to co-functions in particular processes or tissues [51]. Secondly, different combinations of TIR1 and Aux/IAA proteins display a wide range of auxin-binding affinities that are largely determined by the Aux/IAA identity [52]. Thirdly, the Aux/IAA protein family has diversified in degradation and auxin responsiveness, features that are dependent on sequences within and outside of Domain II [53]. Hence, it is necessary to identify the complete set of *Aux/IAA* gene family members within a species, as far as possible, to help develop a comprehensive understanding of *Aux/IAA* biological functions. Although several experimental approaches have been used to characterize gene families in the complex crop amphidiploid *B. napus* in the past [54, 55], genome-wide search based on the available draft genome sequence has provided a more convenient and effective approach for isolation of *B. napus* gene families such as the *SBP-box* and *LEA* families [56, 57]. In this study, we identified all 119 *Aux/IAA* genes present within the genome of *B. napus* using a similar genome-wide search (Fig. 1). This number in *B. napus* represents the largest *Aux/IAA* gene family identified in plants to date, and may have contributed to conferring higher phenotypic plasticity on this crop species.

Tandem duplication and segmental duplication both contribute to the dramatic variation in gene family number and distribution [58]. We mapped all 119 *Aux/IAA* genes on the chromosomes in silico and found five tandemly duplicated gene clusters (Fig. 1). Each of these clusters also represented ancient tandem genes in the *A. thaliana* genome. However, the number of *Aux/IAA* gene family members in *B. napus* is much higher than that in *A. thaliana* (29 members), *B. rapa* (55 members) and *B. oleracea* (60 members). These observations suggest that the expansion of the *B. napus Aux/IAA* gene family may be independent of tandem duplication, and only affected by segmental duplication resulting from *Brassica* WGT and allopolyploidy. The segmental duplication has also led to a much higher number of *Aux/IAA* members in hexaploid bread wheat (84 members) [37] and the palaeopolyploid soybean (63 members) [38]. With 29 *Aux/IAA* members in the *A. thaliana* genome, one would expect ~90 and ~180 *Aux/IAA* genes to be present in the *B. rapa*/*B. oleracea* and *B. napus* genomes. However, only 55, 60 and 119 genes remain in these three genomes, respectively, and the number of genes in the An and Cn subgenomes of *B. napus* is almost the same as that in the diploid progenitors *B. rapa* (Ar genome) and *B. oleracea* (Co genome) (Fig. 1 and Additional file 3). These findings indicate that the loss of *B. napus Aux/IAA* members mainly occurred during the *Brassica* WGT process which resulted in widespread reshuffling of conserved genomic blocks [42, 59], rather than the more recent allopolyploidy from *B. rapa* and *B. oleracea*.

The higher load of *Aux/IAA* genes in *B. napus* increases the probability of divergence within this family. It has been suggested that differentiation of the *Aux/IAA* genes in *A. thaliana* may depend both on molecular properties of proteins as well as expression patterns [60]. In the present study, 43 non-canonical Aux/IAA proteins were found to lack single or multiple domains that may contribute to their divergence (Fig. 3). 35 Aux/IAA proteins did not contain Domain I or Domain III/IV and might have lost capacity in recruiting TPL co-repressors or interaction with ARFs, implying that these proteins could not act as a repressor in auxin signaling, and may function differently in other processes. Notably, most (13/16) of the *Brassica*-specific Aux/IAA genes did not contain Domain I, suggesting that these genes may not contribute to classical auxin signal transduction. In addition, eight Aux/IAA proteins did not contain Domain II. A similar non-canonical Aux/IAA protein lacking domain II has been identified in all other reported plant species. This is consistent with evidence from the *A. thaliana IAA20* which lacks domain II and cannot be rapidly degraded in the presence of basal or increased levels of auxin [53]. Theoretically, such Aux/IAA proteins lacking domain II can repress auxin response gene expression due to the presence of conserved domain I and domain III/IV, and lead to developmental defects similar to *Aux/IAA* dominant mutants in wild-type plants. However, this is not the case in wild-type plants, and some alternative explanations should be sought. One explanation is that these Aux/IAA proteins could interact with additional unknown components and be degraded by a novel process in the auxin signal transduction cascade. Another explanation is that these Aux/IAA proteins have a very low expression level across tissues, and thus little effect on plant growth and development. Within our RNA-seq analysis, we found that the expression level of all eight Aux/IAA genes lacking Domain II was very low in all four major tissues compared with that of the canonical Aux/IAA protein (Additional file 8). Sub-functionalization often depends on changes in the *cis*-regulatory elements of duplicated genes, which primarily leads to a divergence of gene expression [61]. Transcription profiles of these genes showed distinct patterns of expression between different tissues for the *B. napus Aux/IAA* genes possessing the canonical four domains (Fig. 4), which also supports the existence of their sub-functionalization. For example, *BnaC01.IAA.2* and *BnaC01.IAA.6* had much higher expression in stem and flower bud respectively, which suggests that these genes may play a key role in stem and reproductive organ development. This divergence of expression pattern is also observed among homologous *Aux/IAA* genes in *B. napus*. It is noted that a much higher expression divergence was found among homologs resulting from the earlier WGT, although most of the homeologous pairs (An and Cn) from recent allotetraploidy shared similar expression patterns. This difference in functional divergence may be a consequence of the longer time that the older duplicated genes have had to accumulate more changes in promoter regions during their evolutionary history.

In general, orthologs have similar biological function in plant growth and development. In our study, phylogenetic analysis generated 29 orthologous gene sets containing *Aux/IAA* genes from *B. napus*, *B. rapa*, *B. oleracea* and *A. thaliana* (Fig. 2). The biological functions of numerous *Aux/IAA* genes in the model plant *A. thaliana* were well studied mostly by gain-of-function mutations and few by loss-of-function mutations, which can also provide a valuable framework for functional prediction of *Aux/IAA* genes in *Brassica* species. For example, characterization of gain-of-function mutant *iaa16* revealed that *AtIAA16* (*At3g04730*) is involved in root gravitropism and hair development, stem elongation and apical dominance and fertility [25], suggesting that the all *Brassica* Aux/IAA genes in orthologous set 4 may have similar functions in plant development. In contrast, genetic and molecular analysis by loss-of-function mutant *shy2–31* showed that the *IAA3/SHY2* (*At1g04240*) gene is a core factor to balance cell differentiation and division for controlling root meristem size and root growth through interaction between cytokinin and auxin [27], suggesting that the all *Brassica Aux/IAA* genes in orthologous set 5 may have similar role in each species.

In order to unravel whether *Aux/IAA* members in *B. napus* are auxin early-response genes, we analyzed the AuxRE distribution using bioinformatics tools, as well as gene response under experimental auxin treatment. Most *Aux/IAA* genes (85.59%) have an AuxRE within 2 kb upstream of the translation initiation site, similar to observations in other plants [24, 39]. Within our experiment, 34 *Aux/IAA* genes were significantly up-regulated by auxin treatment (Fig. 5b). However, there were a further 28 *Aux/IAA* genes where there was some evidence of partial up-regulation by auxin treatment. This suggests that the expression of *Aux/IAA* genes could be induced by an exogenous auxin signal. In this study, we found that significantly up-regulated genes were enriched in the *Aux/IAA* gene family, having multiple AuxREs in the proximal promoter region. Thus, the number and location of AuxREs may partially account for the differential expression patterns of *Aux/IAAs* under IAA treatment. Moreover, multiple AuxREs near the translation initiation site may be more likely to mediate transcriptional activation of *B. napus Aux/IAA* genes in response to auxin.

## Conclusions

In the present study, a genome-wide analysis of *Aux/IAA* gene family was performed in *B. napus*, which included chromosomal distribution, duplication, phylogeny, gene and protein structure, expression pattern and response to exogenous auxin. 119 *Aux/IAA* genes were identified in the *B. napus* genome by bioinformatic analysis. These genes were non-randomly distributed across the 19 chromosomes and other unassigned scaffolds and symmetrically distributed in the A and C subgenomes. Comparative analysis between *A. thaliana*, *B. napus* and its diploid progenitors *B. rapa* and *B. oleracea* revealed that *Aux/IAA* genes had undergone serious gene loss during *Brassica* WGT but almost unchanged during the recent allotetraploidy in *B. napus*, which resulted in most of the *Aux/IAA* genes having multiple copies. Phylogenetic analysis generated two major groups and seven subgroups. Within these, we identified 29 orthologous gene sets using *A. thaliana Aux/IAA* genes as a reference and two *Brassica*-specific sets. The gene structure was different among all *Aux/IAA* gene members but similar between homologous genes in *B. napus*. Four conserved regions designated as domain I, II, III and IV were identified in *B. napus* Aux/IAA proteins. 63.6% of the proteins contained four canonical domains and other ones lacked one or more domains. Variable expression was observed between *Aux/IAA* genes from different tissues, with the expression pattern distinct among all genes but similar between homeologous pairs from the recent allotetraploidy. The complex motif distribution and expression profiles suggest that the *Aux/IAA* gene family had been subject to sub-functionalization and redundancy in *B. napus*. Furthermore, 34 genes enriched with multiple AuxREs in their promoter proximal regions, could be significantly up-regulated by exogenous auxin treatment in seedling. The results presented in this study will be useful for future functional dissection of *Aux/IAA* genes in *B. napus*.

## Additional files

Additional file 1: List of primers for qRT-PCR in this study. (XLS 21 kb)

Additional file 2: Information of *Aux/IAA* genes identified in *B. napus*. (XLS 51 kb)

Additional file 3: The orthologous *Aux/IAA* gene sets among *B. rapa* (Ar genome), *B. oleracea* (Co genome), *B. napus* (An and Cn genomes) and *A. thaliana.* (XLS 53 kb)

Additional file 4: Comparison of orthologous *Aux/IAA* gene structure among *A. thaliana* and *B. rapa*, *B. oleracea*, *B. napus*. The 29 orthologous sets and two *Brassica* specific sets are indicated with colored lines. (PDF 5964 kb)

Additional file 5: Multiple sequence alignment of *Aux/IAA* genes in *B. napus*. Four conserved domains are indicated with black boxes. LxLxLx and GWPPv/i motifs in domain I and II are highlighted with black lines. The second LxLxLx motif between domain I and II is indicated with red boxes. NLSs and βαα motif are represented by black solid rectangles. The PB1 domain features of a conserved lysine and the OPCA-like motif phosphorylation sites are emphasized by black arrows (Korasick et al., *Proc Natl Acad Sci USA,* 2014(111): 5427–5432). (PDF 9716 kb)

Additional file 6: Comparison of orthologous Aux/IAA protein domain among *A. thaliana* and *B. rapa*, *B. oleracea*, *B. napus*. The 29 orthologous sets and two *Brassica* specific sets are indicated with colored lines. (PDF 4357 kb)

Additional file 7: Gene ontology of *B. napus Aux/IAA* genes. (PDF 2622 kb)

Additional file 8: FPKM value of *B. napus Aux/IAA* genes in four major tissues. (XLS 37 kb)

Additional file 9: qRT-PCR validation of expression pattern of six *Aux/IAA* genes. (PDF 1032 kb)

## Abbreviations

ARF: Auxin response factor
Aux/IAA: Auxin/Indoleacetic acid
AuxRE: Auxin response element
CD: Conserved domain
CDS: Coding domain sequence
DBD: DNA binding domain
DEG: Differential expression gene
FPKM: Fragments per kilobase per million mapped reads
GRAVY: Grand average of hydropathicity
GSDS: Gene structure display server
HMM: Hidden markov model
MS: Murashige and Skoog
MYA: Million years ago
NJ: Neighbor-joining
NLS: Nuclear localization signal
ORF: Open reading frame
TIR1/AFB: Transport inhibitor response1/Auxin signaling F-Box
WGT: Whole genome triplication
