# Supplementary figures and images for: Genome-wide analysis of the auxin/indoleacetic acid (Aux/IAA) gene family in allotetraploid rapeseed (Brassica napus L.)
Source: BMC Plant Biol. 2017 Nov 16;17:204. doi: 10.1186/s12870-017-1165-5 (PMC5691854; doi:10.1186/s12870-017-1165-5)

GroupA1

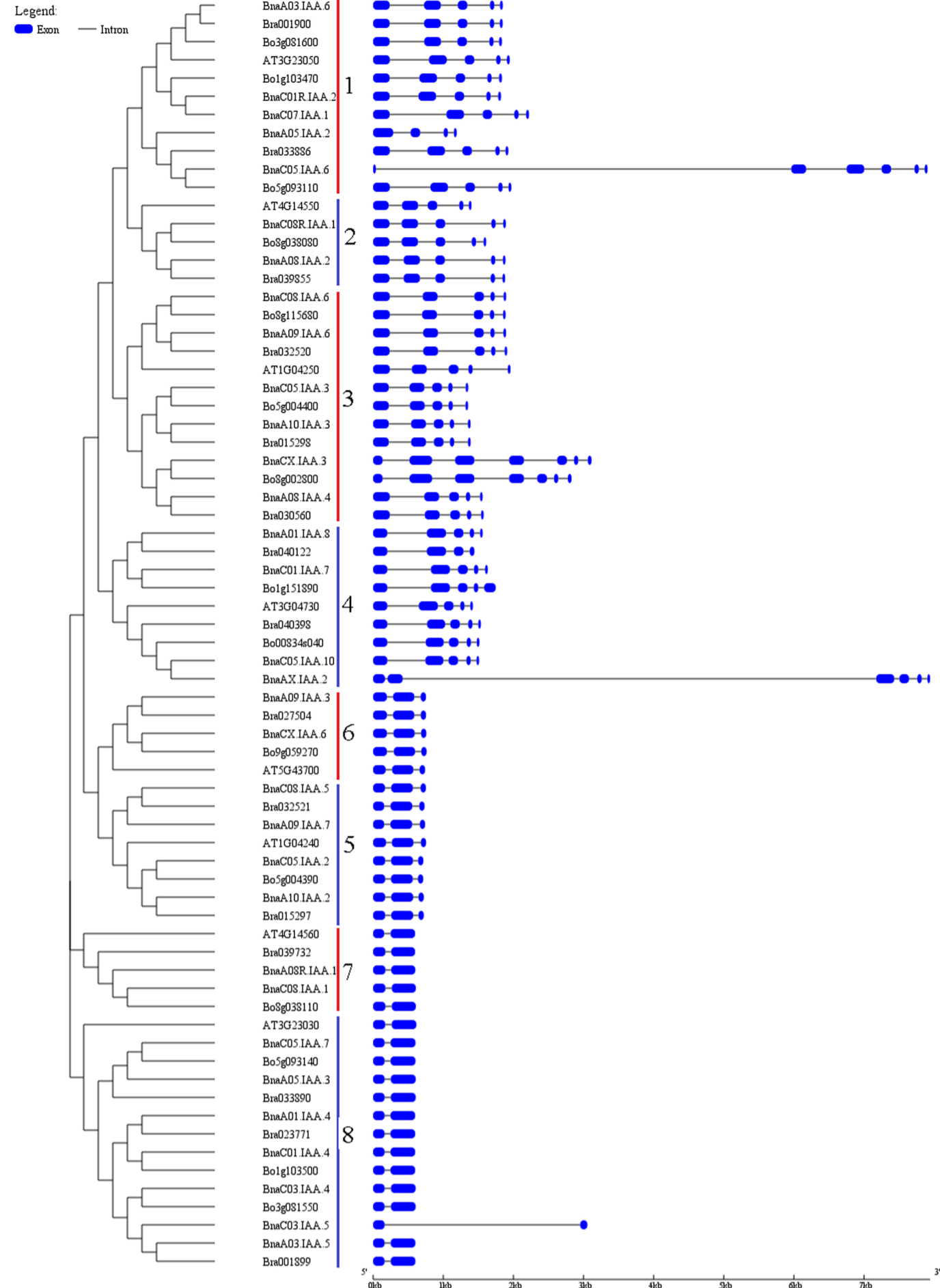

GroupA2

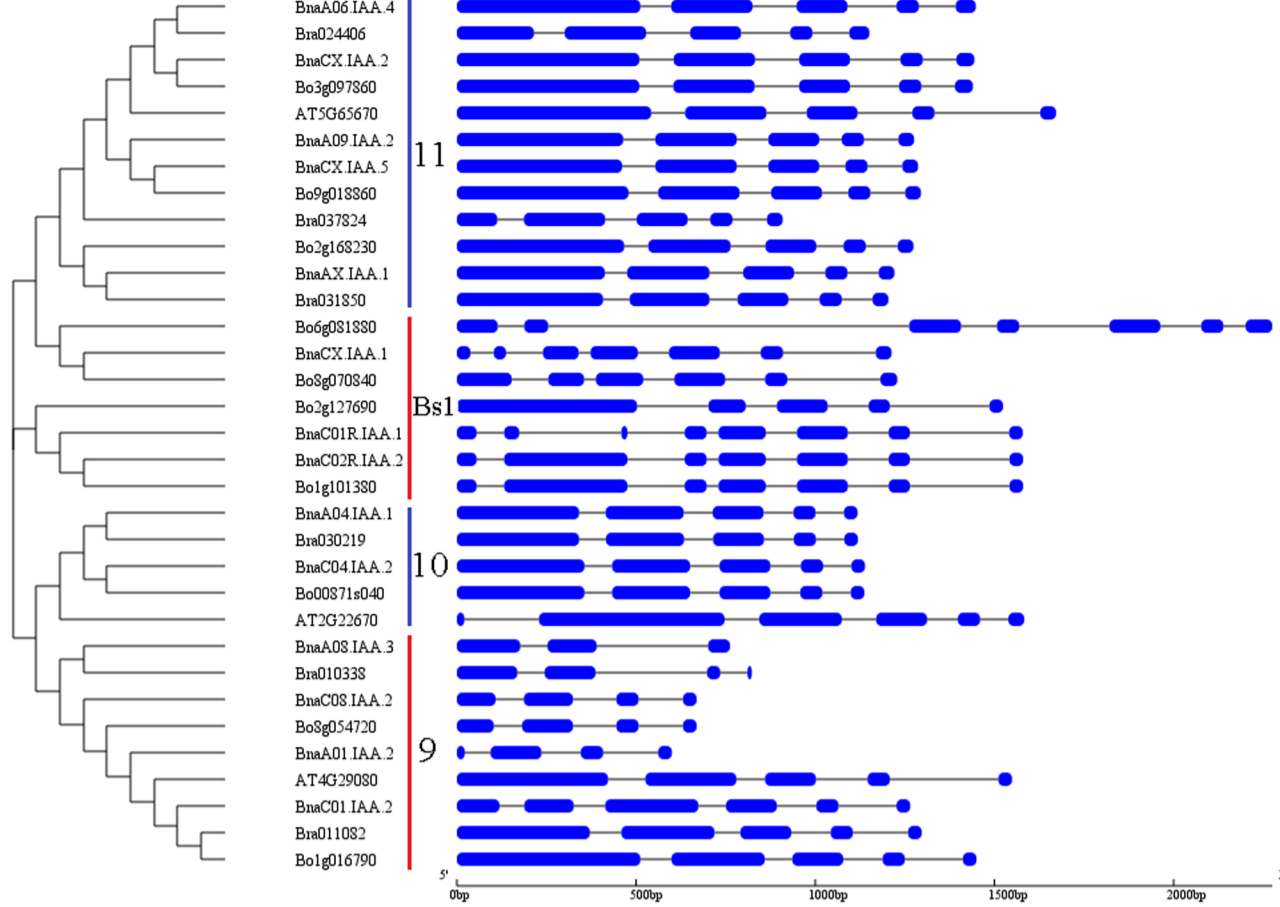

GroupA3

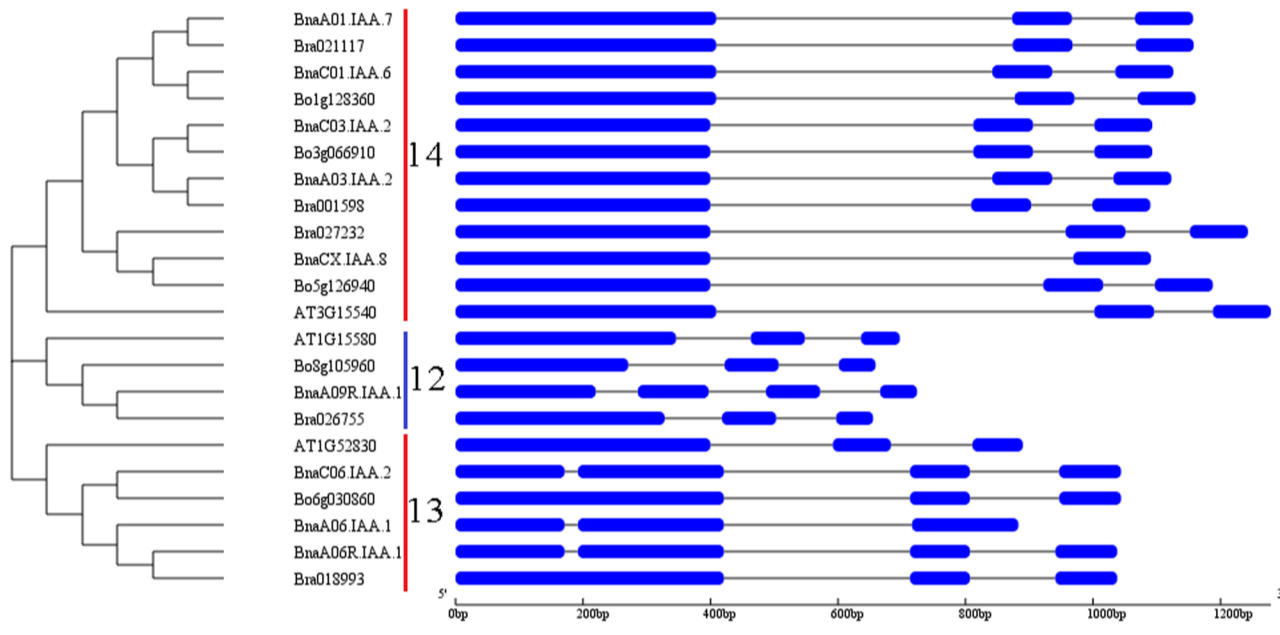

GroupB1

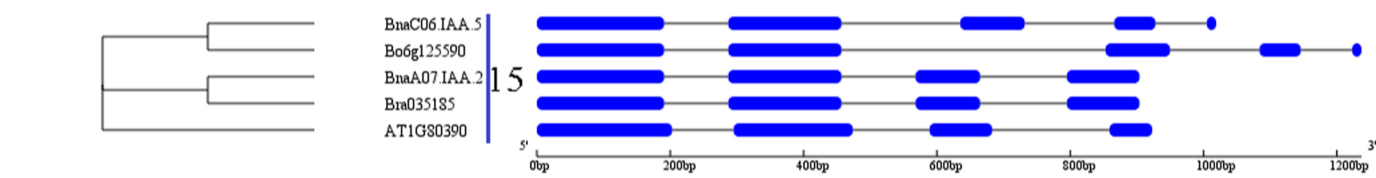

GroupB2

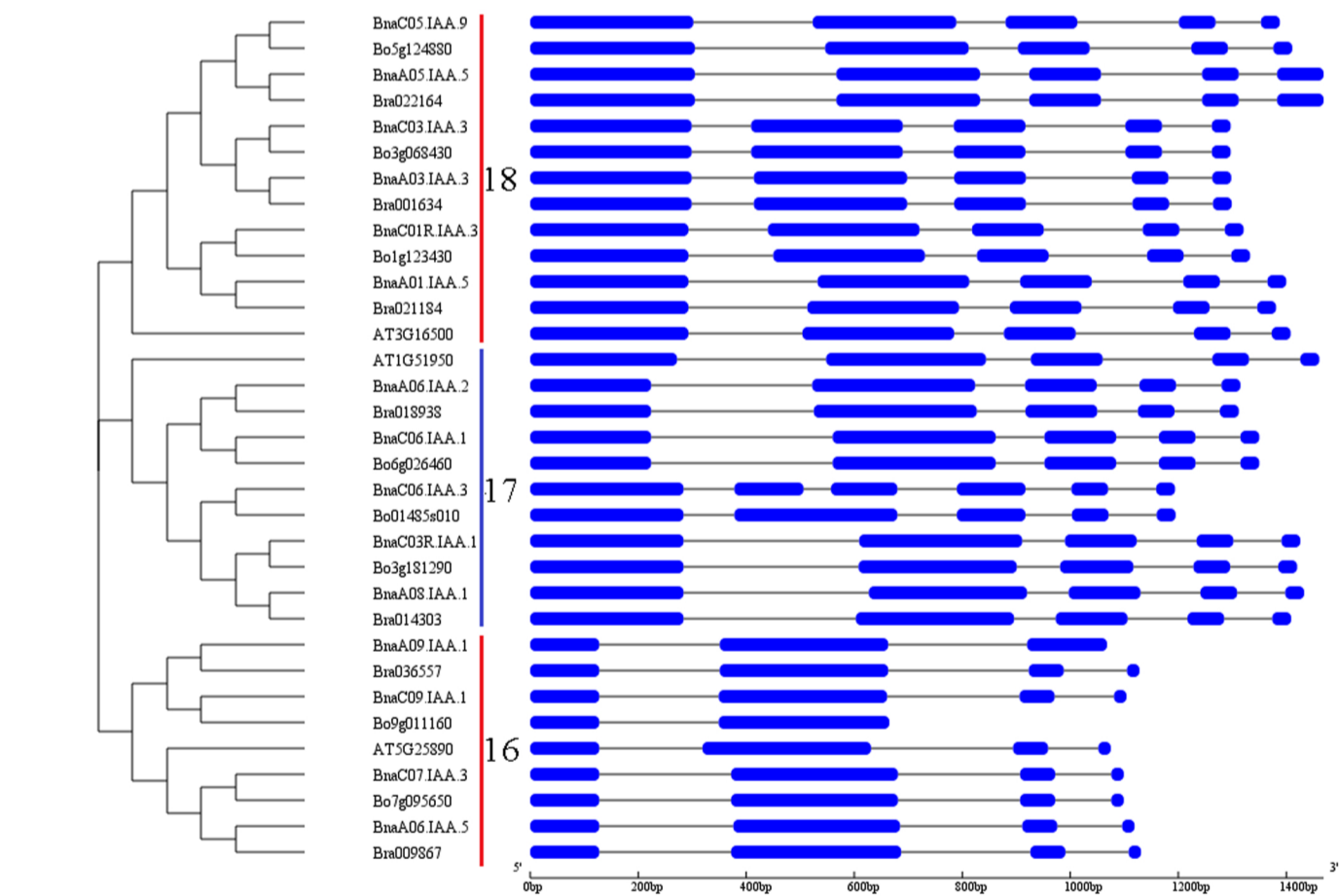

GroupB3

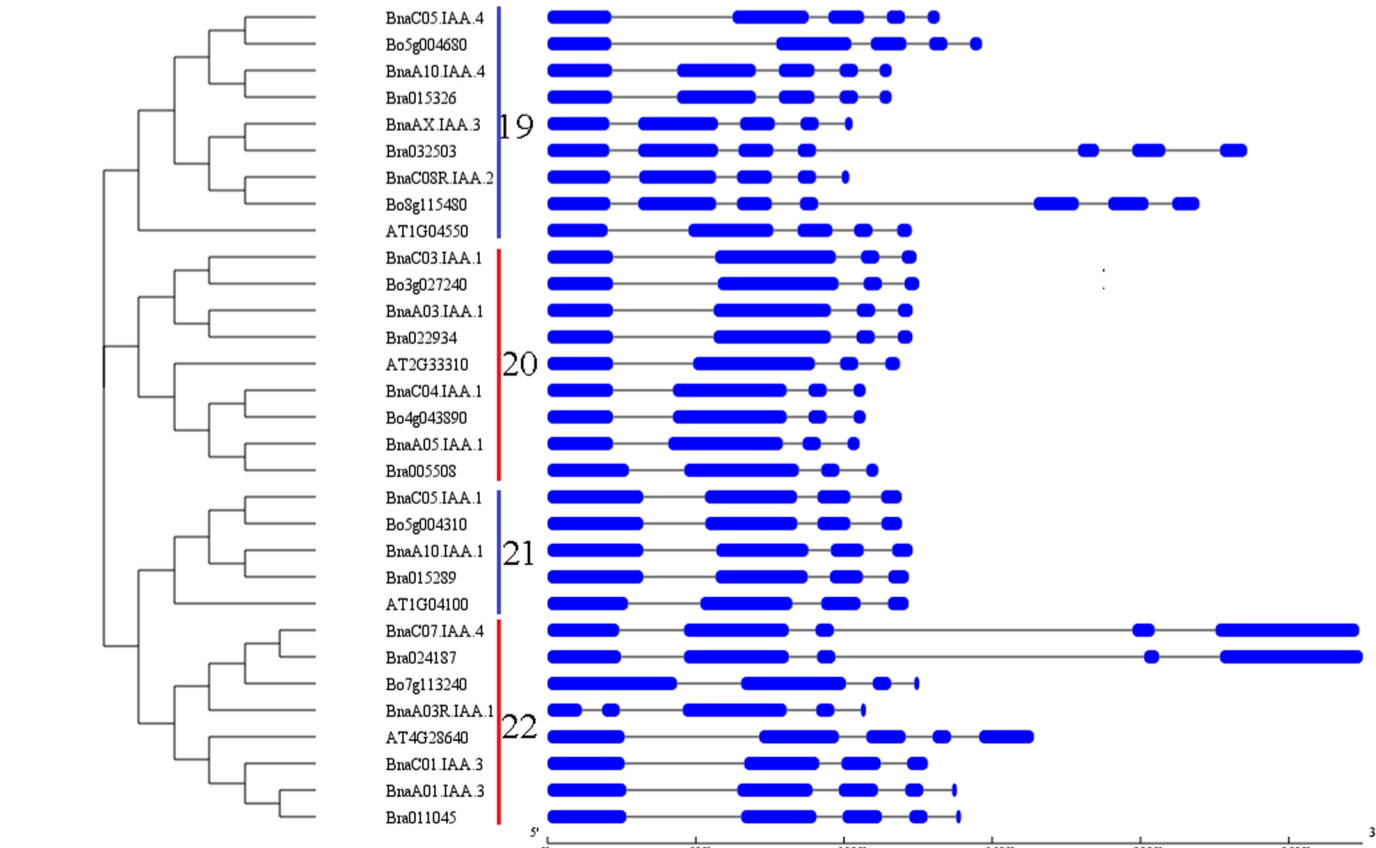

GroupB4

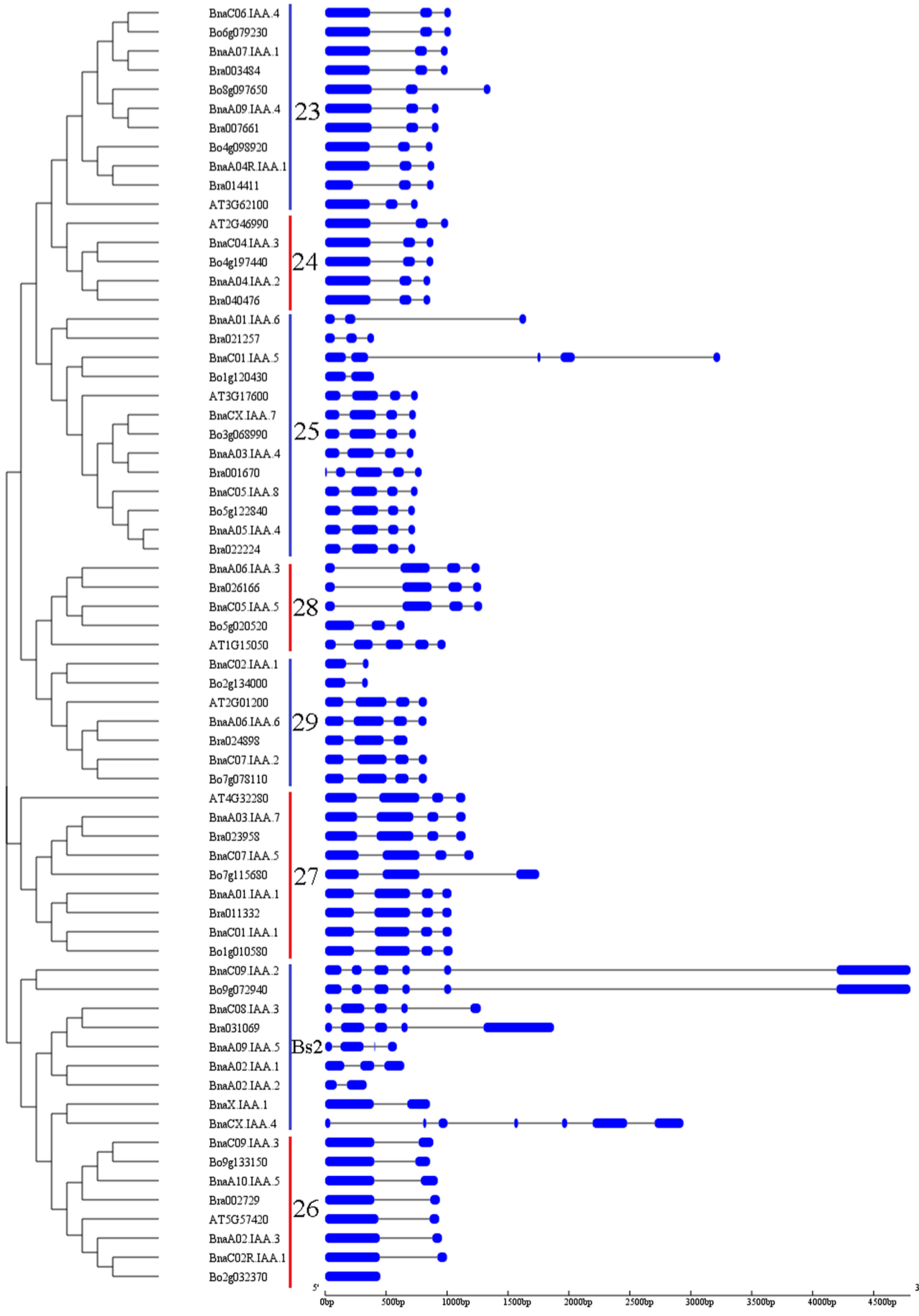

Supplement: Supplementary file 4 — Comparison of orthologous Aux/IAA gene structure among A. thaliana and B. rapa, B. oleracea, B. napus. The 29 orthologous sets and two Brassica specific sets are indicated with colored lines. (PDF 5964 kb) [file 12870_2017_1165_MOESM4_ESM.pdf]

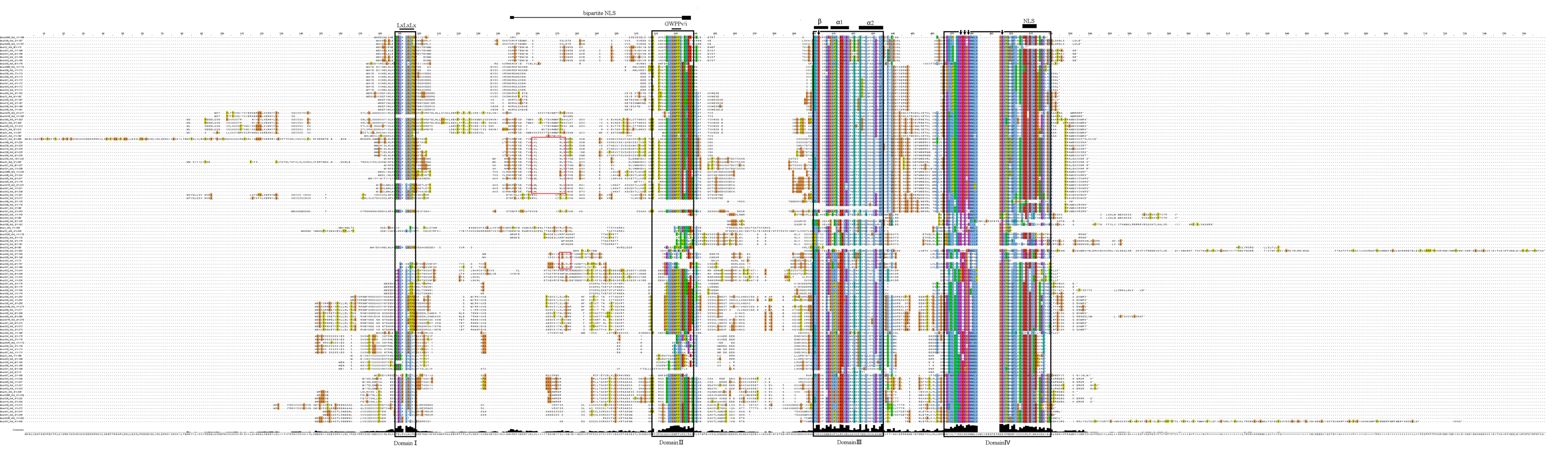

Supplement: Supplementary file 5 — Multiple sequence alignment of Aux/IAA genes in B. napus. Four conserved domains are indicated with black boxes. LxLxLx and GWPPv/i motifs in domain I and II are highlighted with black lines. The second LxLxLx motif between domain I and II is indicated with red boxes. NLSs and βαα motif are represented by black solid rectangles. The PB1 domain features of a conserved lysine and the OPCA-like motif phosphorylation sites are emphasized by black arrows (Korasick et al., Proc Natl Acad Sci USA, 2014(111): 5427–5432). (PDF 9716 kb) [file 12870_2017_1165_MOESM5_ESM.pdf]

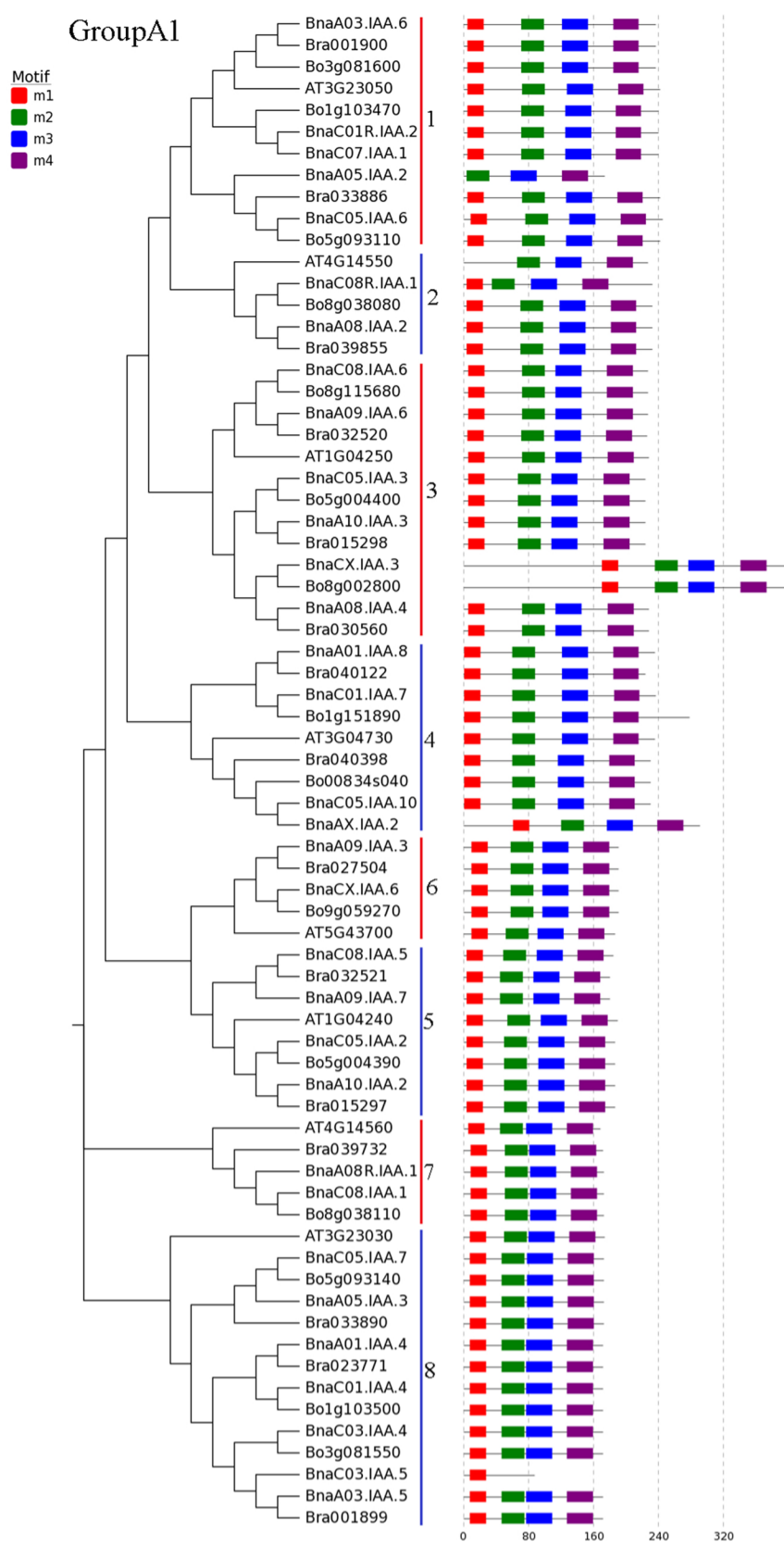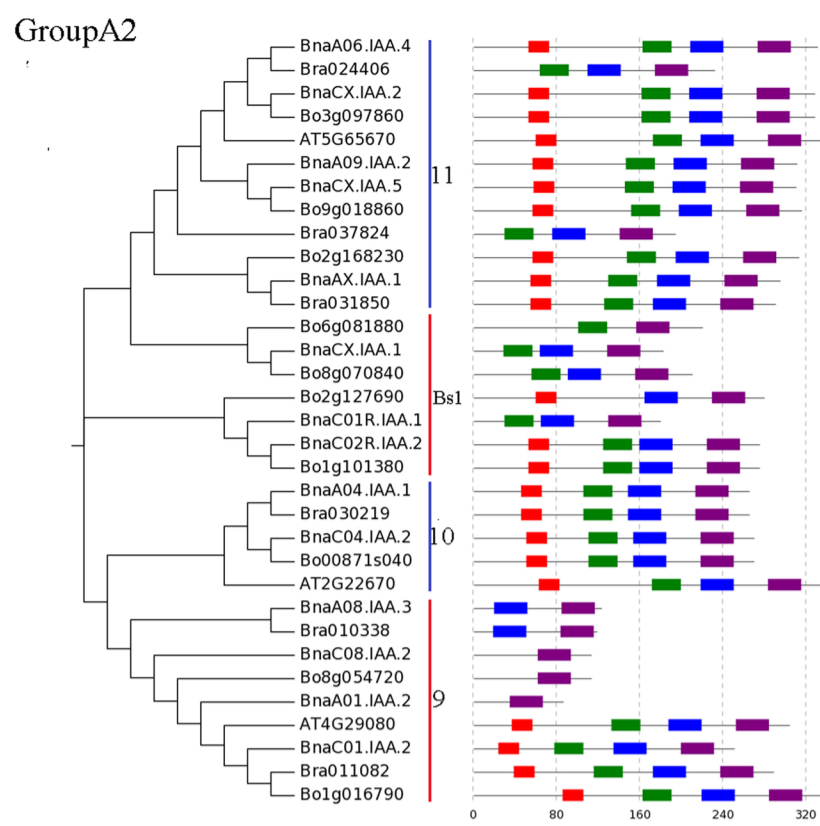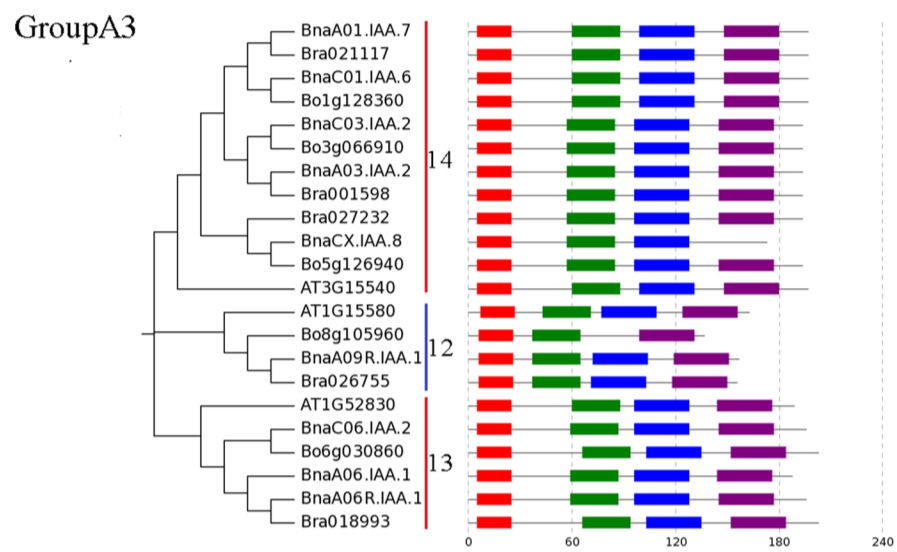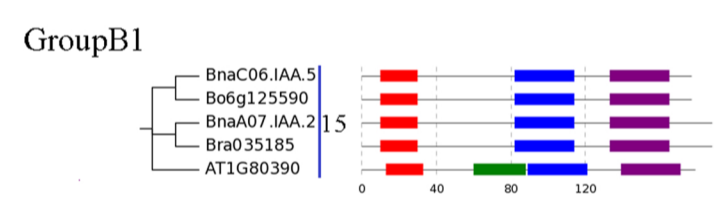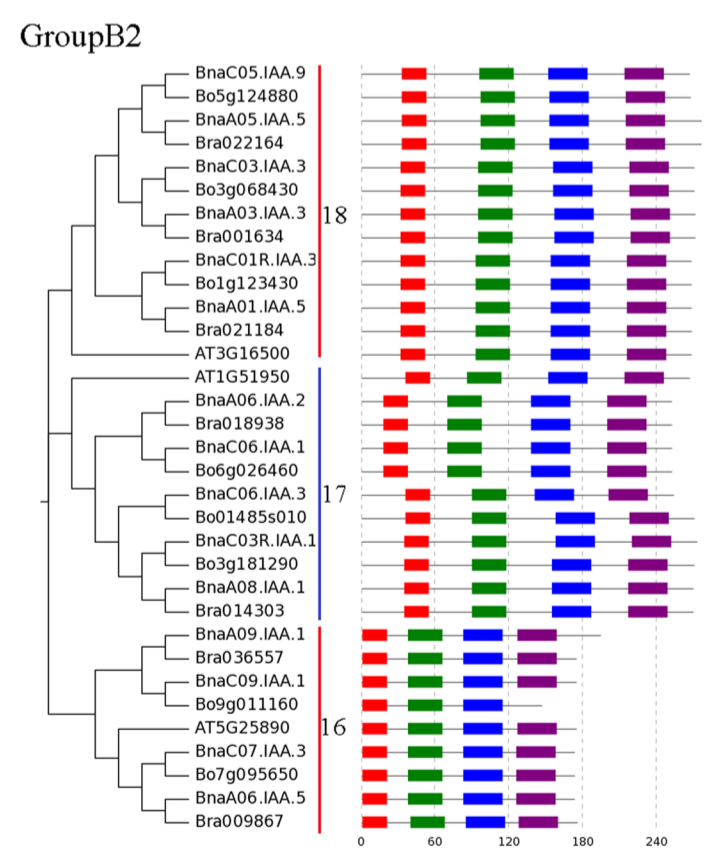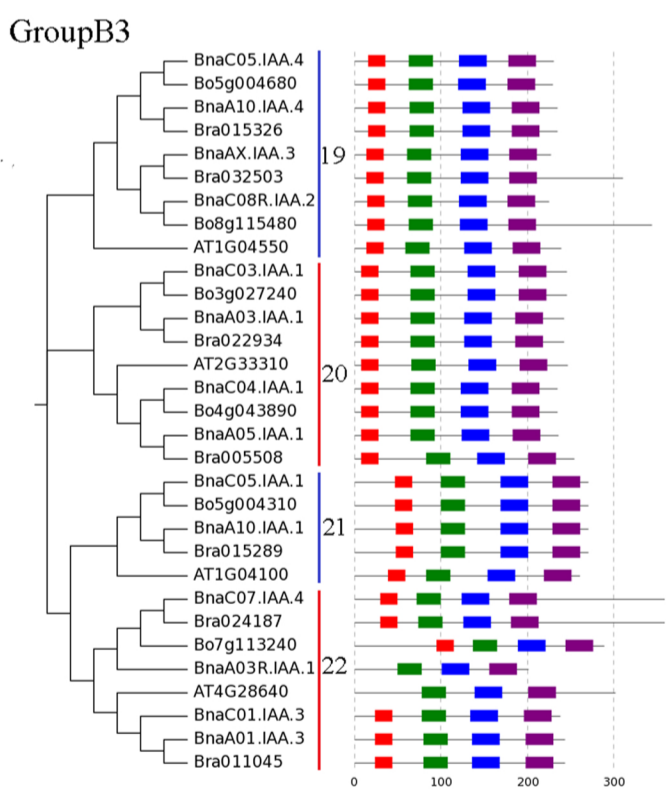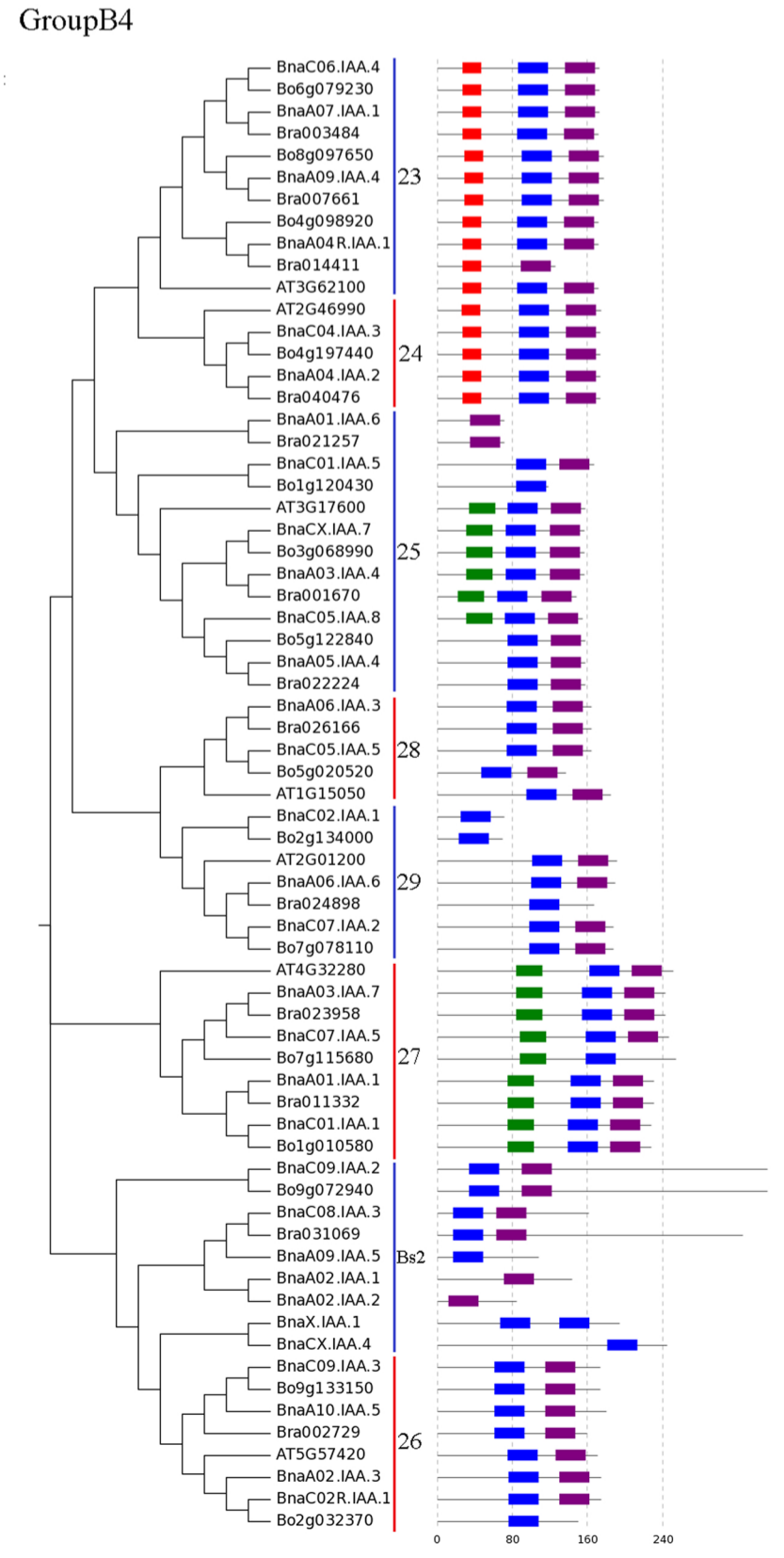

Supplement: Supplementary file 6 — Comparison of orthologous Aux/IAA protein domain among A. thaliana and B. rapa, B. oleracea, B. napus. The 29 orthologous sets and two Brassica specific sets are indicated with colored lines. (PDF 4357 kb) [file 12870_2017_1165_MOESM6_ESM.pdf]

A

### Cellular Component

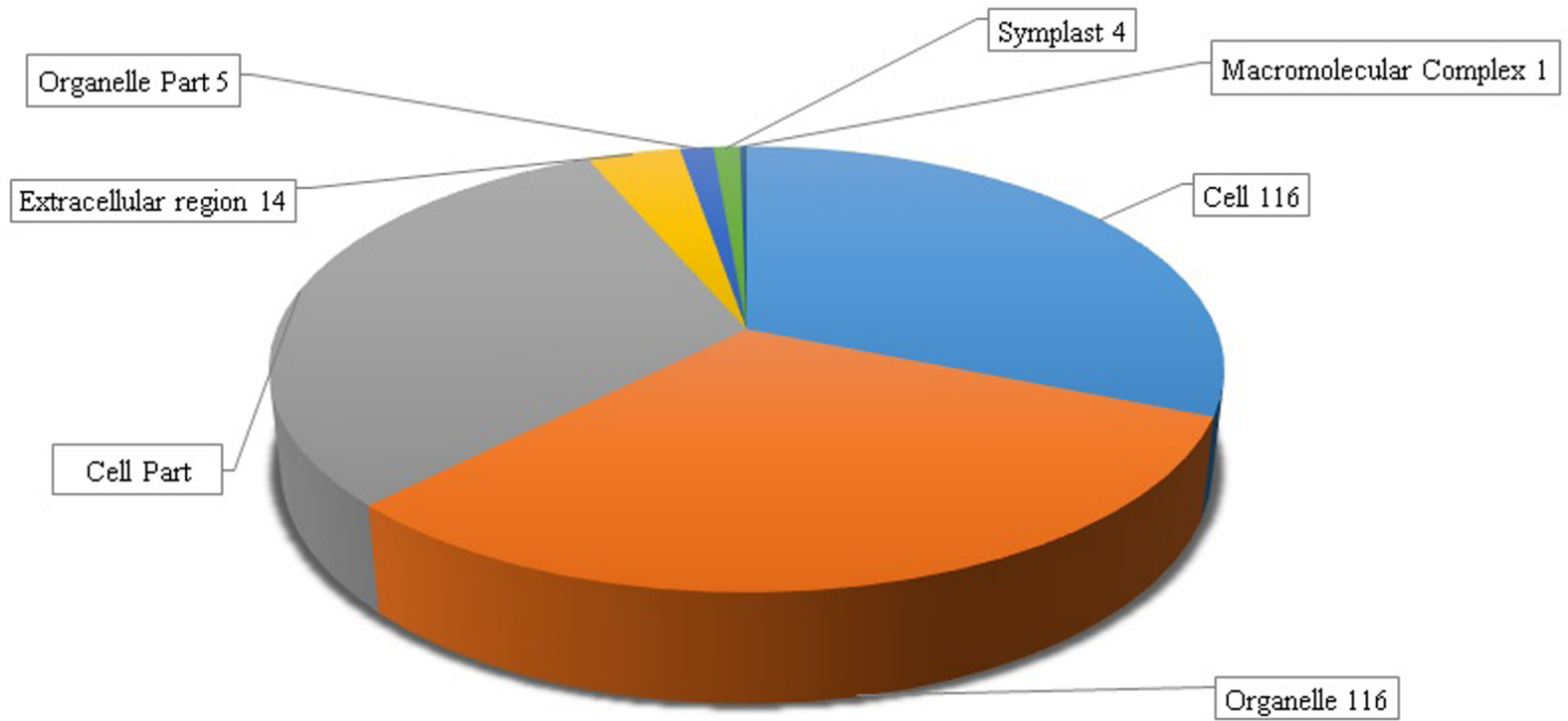

B

### Biological Process

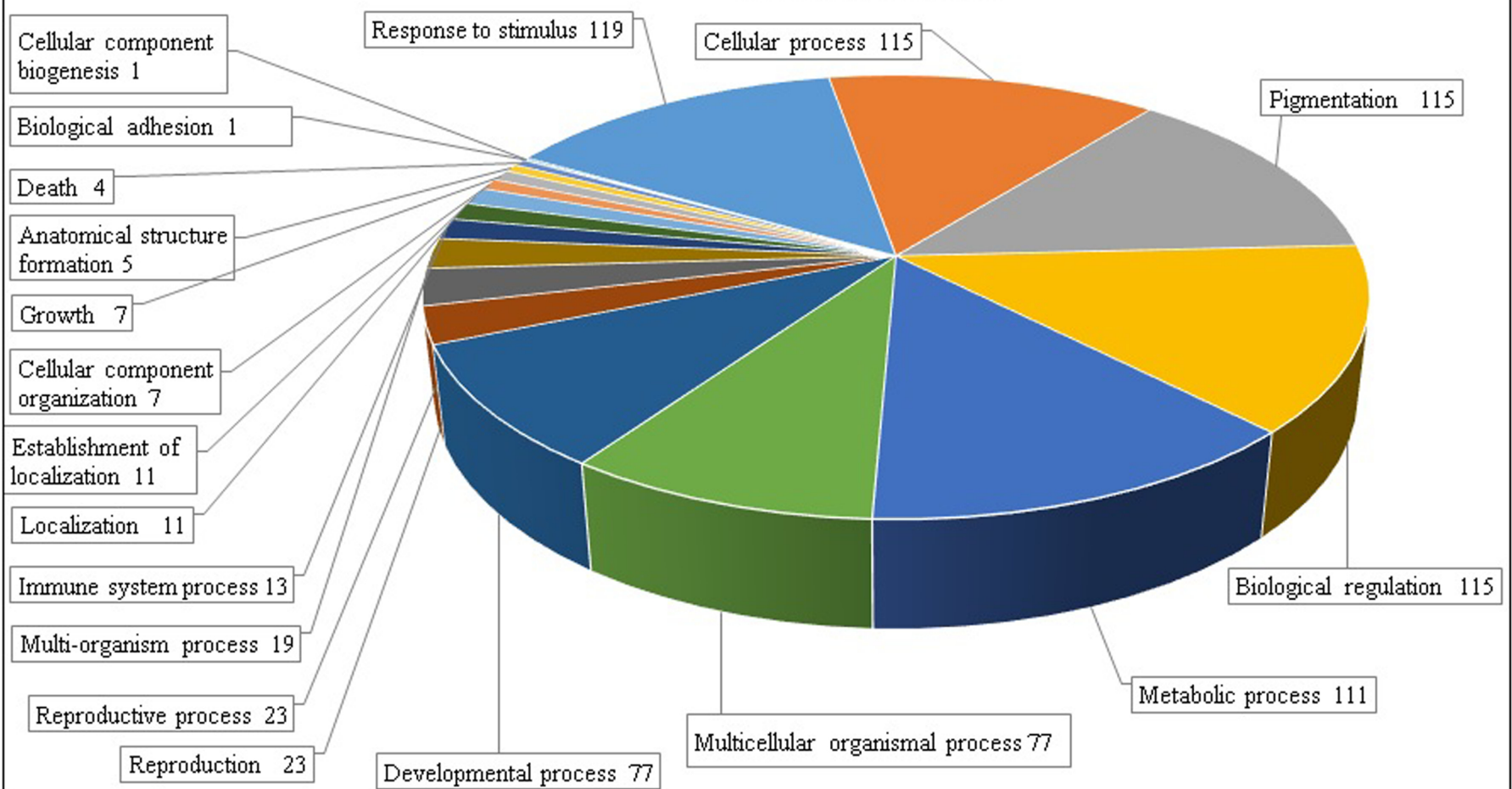

C

### Molecular Function

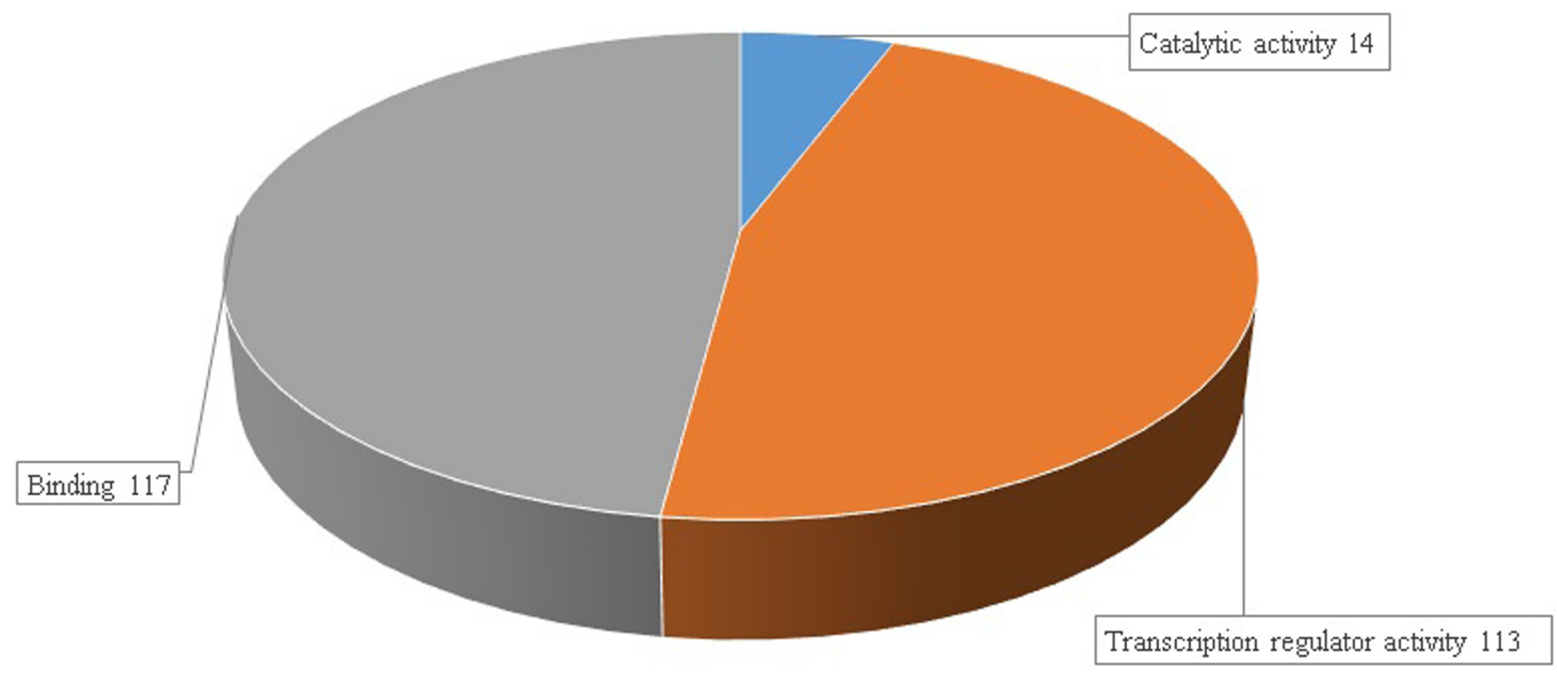

Supplement: Supplementary file 7 — Gene ontology of B. napus Aux/IAA genes. (PDF 2622 kb) [file 12870_2017_1165_MOESM7_ESM.pdf]

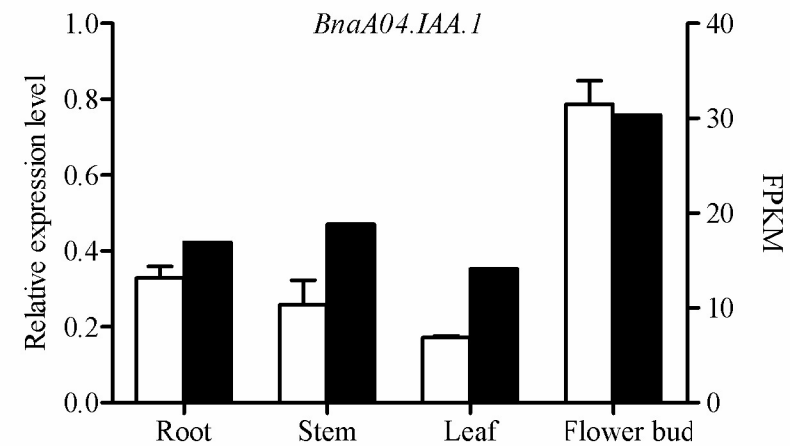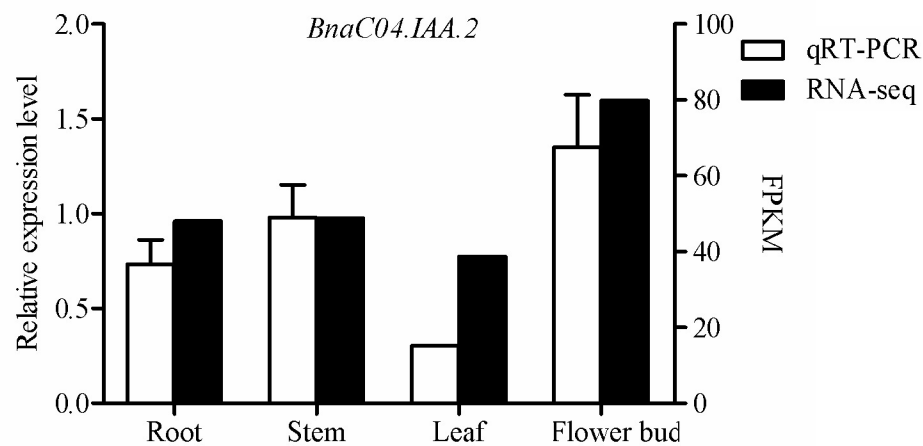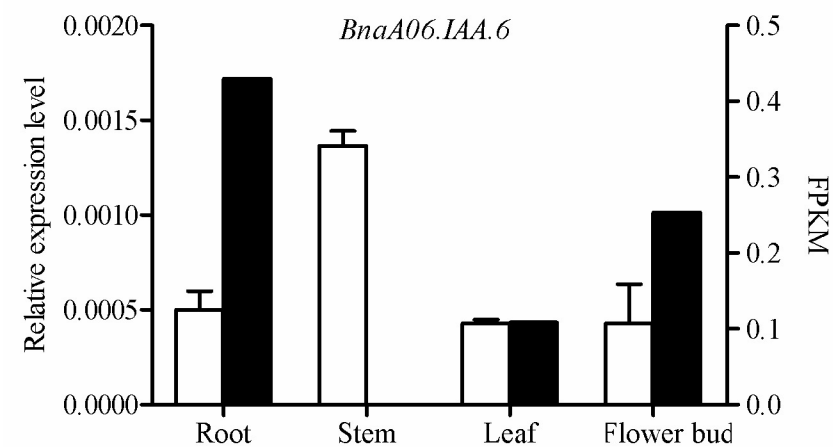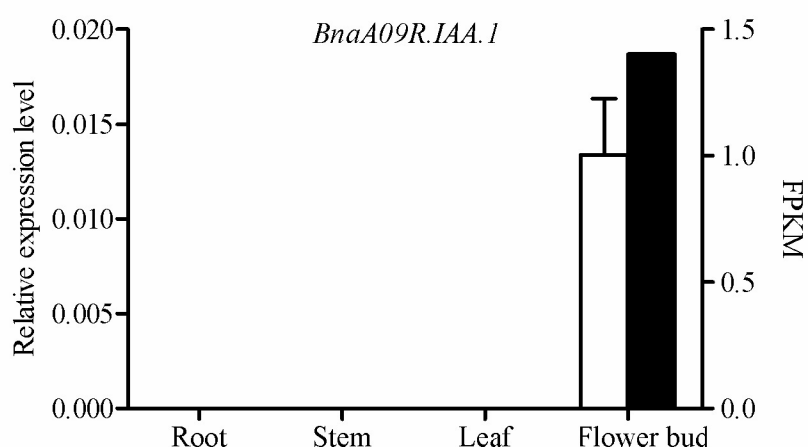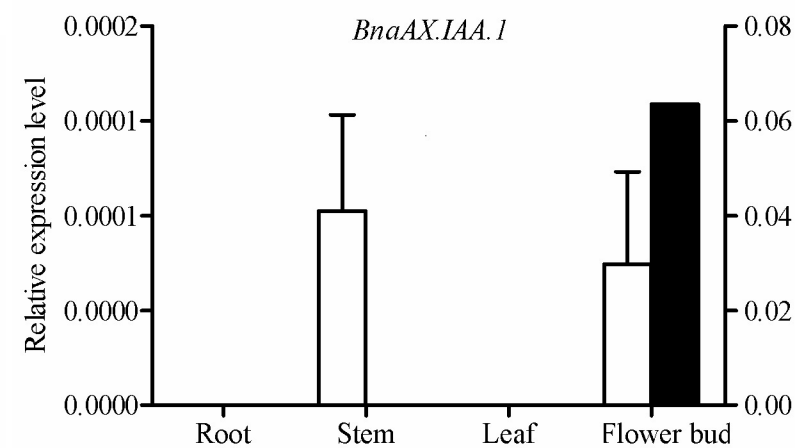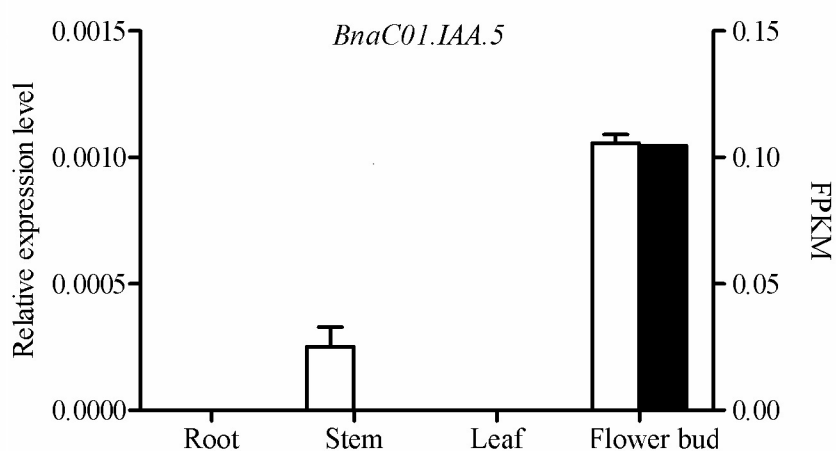

Supplement: Supplementary file 9 — qRT-PCR validation of expression pattern of six Aux/IAA genes. (PDF 1032 kb) [file 12870_2017_1165_MOESM9_ESM.pdf]
